# Riverine heat waves on the rise, outpacing air heat waves

**DOI:** 10.1073/pnas.2503160122

**Published:** 2025-09-22

**Authors:** Kayalvizhi Sadayappan, Li Li

**Affiliations:** ^a^Department of Civil and Environmental Engineering, The Pennsylvania State University, University Park, PA 16802

**Keywords:** aquatic ecosystems, climate change, climate extremes, heat waves, deep learning

## Abstract

While air heat waves often grab headlines, riverine heat waves have gone quietly unnoticed because rivers are commonly perceived as cool refuges. Analysis of riverine heat waves has been hindered by fragmented datasets, despite a proliferation in water-temperature monitoring with sensors and satellites. Here, we analyze riverine heat wave events by training one single deep learning (long short-term memory) model and reconstructing consistent and continuous daily water temperatures (WT) in 1471 sites in the Contiguous United States (1980–2022). We show that riverine heat waves occur at about half the frequency (2.3 versus 4.6 events/year), a third intensity (2.6 versus 7.7 °C/event), but almost double the duration (7.2 versus 4.0 d/event) of air heat waves. Riverine heat wave events have increased at double to quadruple rates of air heat wave events, amounting to an additional 1.8 events/year in frequency, 0.43 °C/event in intensity, 3.4 d/event in duration, and 7 to 15 additional thermal stress days for aquatic ecosystems in 2022 compared to 1980. Rising riverine heat waves have outpaced those of air heat waves in 65 to 76% of the sites, particularly in regions experiencing accelerated warming (e.g., the Rockies). Riverine heat wave trends are driven predominantly by climate-induced changes such as warming and dwindling snowpacks and water flow. Human activities do play important roles: large dams elongate, whereas agriculture reduces heat waves. These results highlight anthropogenic climate change as the primary external driver, whereas human-induced structural changes as the secondary internal modulators of river response to heat disturbance. The widespread rise of riverine heat waves threatens aquatic ecosystems and water-energy-food security, underscoring the need for their global characterization and risk assessment.

## Significance

Riverine heat wave events—periods of abnormally high riverine water temperatures (WT)—can substantially impair aquatic ecosystems, water quality, and food and energy production. However, comprehensive analysis of riverine heat waves is still emerging, long hindered by fragmented and discontinuous WT data. Here, we used a deep learning model and reconstructed consistent daily WT in 1471 U.S. river sites (1980–2022). Results show that riverine heat wave events occur less frequently and intensively but last nearly twice as long as air heat waves. Alarmingly, riverine heat waves have risen at much faster rates than air heat waves. Results here underscore the need for coordinated monitoring and data consolidation efforts for riverine heat waves, and their incorporation into global climate risk assessment and adaptation policies.

Heat waves are anomalous events when temperatures remain high over extended periods of time. Air heat waves have occurred increasingly in recent years, causing heavy societal and economic losses (1, 2). Riverine heat waves, however, have gone quietly unnoticed because rivers are often perceived as cool refuges. Riverine heat waves disrupt aquatic life and have been linked to mortality (3–5), growth disorders, and reproductive impairments (6). These effects can cascade through populations and jeopardize food supplies such as salmon and trout (7–10). Riverine heat waves can additionally degrade water quality (11–13), increase water treatment cost (14), and impair energy production in thermoelectric and nuclear power plants by reducing water cooling capacity (15).

Heat waves in air, lakes, and oceans have been extensively studied and are known to increase in intensity, duration, and frequency (16–19). Although river water temperatures (WT) have been shown to rise more rapidly than oceans and slower than lakes (20), riverine heat waves are only beginning to receive attention (21). WT in large water bodies such as lakes and oceans can be estimated reliably and consistently using satellite data, similar estimation for flowing inland waters has been limited to large rivers (22, 23). Narrow, meandering streams constitute more than 70% of global river networks (24, 25) but often escape the spatial resolution of satellite observations (22, 26). While in-stream and air-borne thermal sensor technologies have proliferated since 1990s, the efforts of compiling, organizing, and curating scattered WT records have not kept pace (27), resulting in temporally inconsistent and spatially fragmented data. As an example, Tassone et al. (21) reported, for the first time, increasing frequency of riverine heat wave events across 70 US rivers (21); however, limited data yielded estimation of insignificant trends in many sites. In general, the lack of consistent and continuous data has hindered the identification and characterization of riverine heat wave events across diverse climate and land use conditions such that they have remained largely unexplored.

WT and the development of riverine heat waves depend on an array of energy and water exchange processes such as heat transfer at river–atmosphere and river–land interfaces, which further depend on external climate drivers and internal catchment structures (28). Increasing air temperature (AT) has been identified as elevating WT from local to global scales (20, 29). Various aspects of hydrology, including diminishing snowpack, river discharge, and groundwater inflow, have also been recognized as influential (21, 30, 31). Hydrological processes are driven not only by climate but also by vegetation, geology, and human-induced infrastructure such as agriculture, urbanization, and dam construction (32, 33). In general, it has remained equivocal whether climate change or human factors predominantly drive WT and riverine heat waves.

Here, we ask the questions: 1) How do the characteristics and trends of riverine heat waves, specifically intensity, duration, and frequency, compare with those of air heat waves? 2) Which factor drives the rising trends of riverine heat wave more: anthropogenic climate change or direct human activities? We overcome the challenge of data limitations by employing a deep learning long short-term memory (LSTM) model, a type of recurrent neural network known for leveraging diverse, inconsistent observations to reconstruct continuous time series data (34–36). We compiled data from 1,471 sites in US rivers (1980–2022) with mean percentage of temporal data coverage of 21.7% (*SI Appendix*, Fig. S1). One single LSTM model was trained to fill data gaps and predict daily WT and discharge in all sites using static catchment attributes and time series of daily meteorological data as input (*SI Appendix*, Table S1). We additionally identified the most influential drivers of riverine heat wave trends using a Boosted Regression Tree (BRT, see Methods) model (37).

## Results

### Robust Model Performance.

**Fig. 1.**
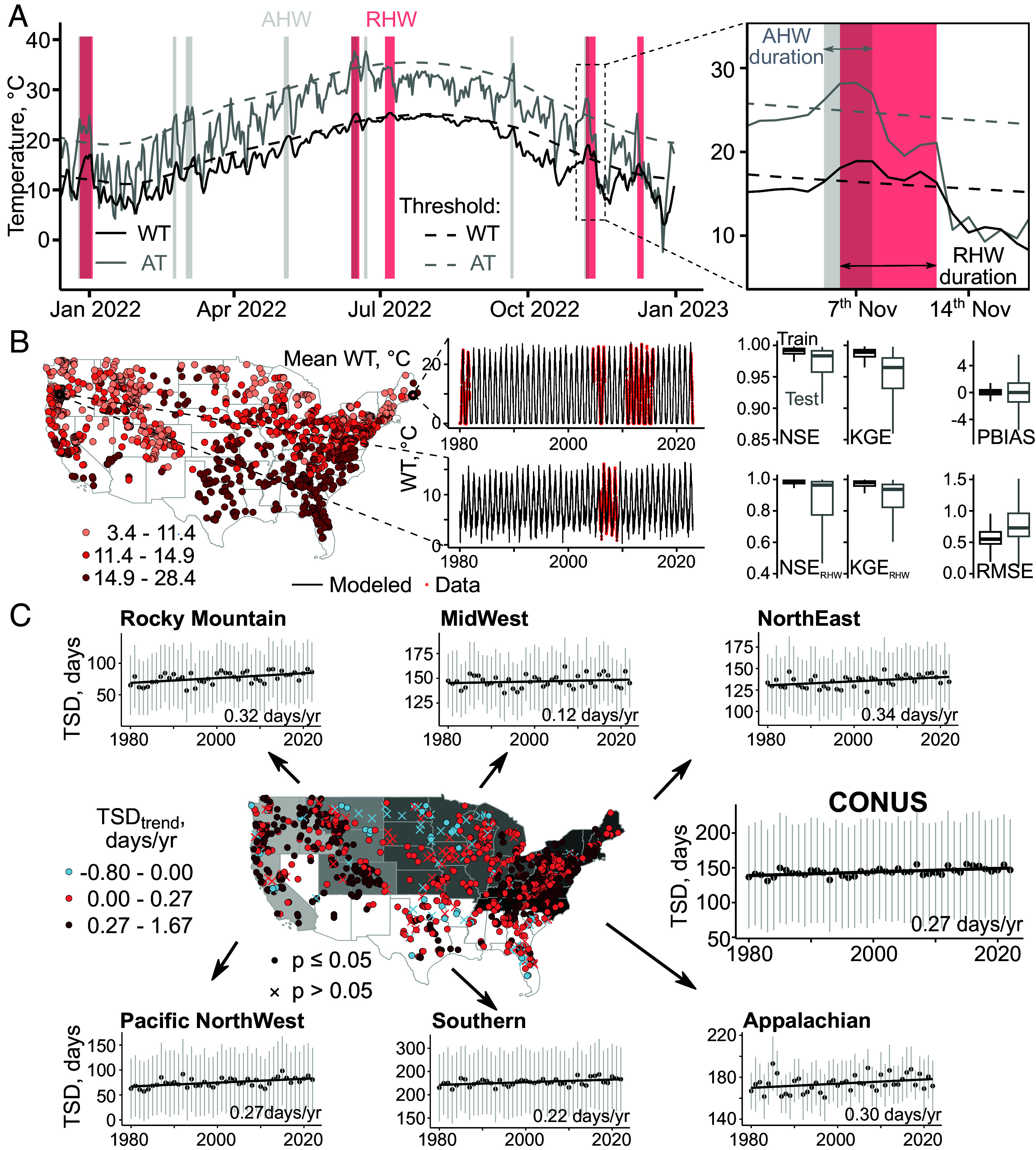
heat wave events, deep learning model, and thermal stress days. (*A*) Illustration of air heat wave (AHW, gray band) and riverine heat wave (RHW, light red band) events using daily maximum air temperature (AT) and daily water temperature (WT), respectively, in a representative site (USGS 02204130). Air and riverine heat wave events are defined as occurring when AT and WT exceed their corresponding seasonally varying 90th percentile thresholds (dashed gray and black lines) for at least 3 and 5 d, respectively. (*B*) *Left*: site map of long-term mean WT; *Middle*: observed (red) and modeled (black line) WT in two representative sites (USGS 01022500 and 14181750; *Right*: model performance metrics (25th, 50th, and 75th percentiles) for NSE, KGE, PBIAS, and RMSE over training (black) and testing (gray) periods (*Right*) and during events (NSE_RHW_ and KGE_RHW_). (*C*) Map of trends in annual thermal stress days (TSD, WT > 15 °C); only sites with at least 10 annual TSD are shown. Side figures show time series of annual TSD in regions (defined in *SI Appendix*, Text S1) and in CONUS. Circles and error bars are mean and one SD, respectively.

The trained model has good performance based on common metrics including Nash-Sutcliffe efficiency (NSE) and Kling-Gupta efficiency (KGE), percent bias (PBIAS) and Root Mean Square error (RMSE). Best performing models mean close-to-one NSE and KGE values, low RMSE and close-to-zero PBIAS values. Here, the median NSE values are 0.99 and 0.98, and KGE values are 0.99 and 0.96 for training and testing periods, respectively (Fig. 1), which indicates near-perfect data reconstruction and far exceeds the commonly used satisfactory value of 0.5. The PBIAS values are 0.1% and 0%, and RMSE values are 0.55 °C and 0.73 °C during the training and testing periods, respectively, indicating minimal model bias and errors. Although most models underperform during extreme events, the trained model here performs similarly well during heat waves. For daily discharge, the model also has good performance with median NSE, KGE, PBIAS, and RMSE values of 0.75, 0.71, -8.6%, and 5.0 m^3^/s, respectively, during testing periods (*SI Appendix*, Fig. S2). Among the 1,471 sites, 1,276 sites have NSE and KGE values exceeding 0.8, low absolute values of PBIAS below 25% and RMSE within the 0.16 to 3.06 °C range during the testing period. These well-performing sites were used for further heat wave analysis.

### Pervasive Rise of Thermal Stress Days.

Modeled WT highlights increasing trends of thermal stress days for aquatic life. Most aquatic species are cold-blooded, with optimal temperatures varying by species, populations, and life stages (38). Some species tolerate up to 20 °C, but sensitive species like bull trout prefer waters below 15 °C (39). Here, we define “thermal stress” and “critical thermal stress” days as those with WT above 15 and 20 °C, respectively. The annual thermal stress days have increased by 11.6 d on average across the CONUS (1980–2022, Fig. 1*C* and *SI Appendix*, Text S1), increasing most rapidly in the Northeastern region (0.34 d/yr), followed by the Rocky Mountains (0.32), and the Appalachian region (0.30). These trends amount to an average increase of 14.6, 13.8, and 12.9 d in 2022 compared to 1980 in these regions, respectively—far exceeding the increase in the Midwest region (5.2 d). The critical thermal stress days show different patterns, with most rapid increases in the Southern and Appalachian regions (15.1 to 16.3 d) compared to the Midwest region (*SI Appendix*, Fig. S3). The Northeastern and Rocky Mountain regions, with typical WT below 20 °C, have witnessed slower (0.24 d/y) increase in critical thermal stress days. Thermal stress and critical thermal stress days have increased significantly (*p* ≤ 0.05) in 82 and 74% of the 1,471 sites, respectively.

### Less Frequent and Intensive But Longer Riverine heat waves Compared With Air heat waves.

**Fig. 2.**
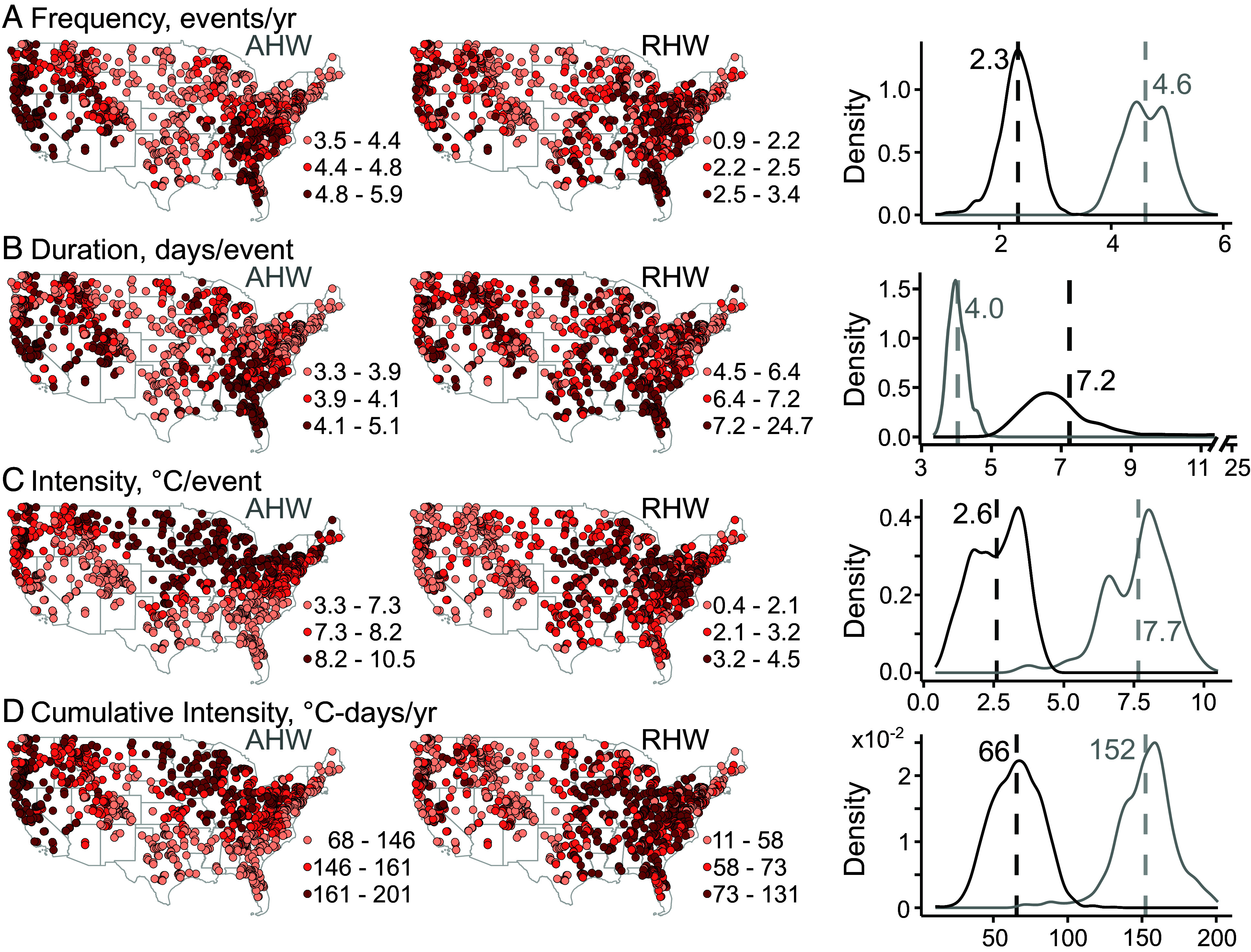
Spatial and statistical distribution of mean annual characteristics of air heat wave (AHW) and riverine heat wave (RHW) events for the best-performing 1,276 sites (1980–2022). Annual (*A*) frequency, the number of heat wave events in a year. (*B*) duration, average duration of all events in a year (zero if no event occurred). (*C*) intensity, average intensity of all events in a year. (*D*) cumulative intensity, the sum of the products of duration and intensity of all events in a year. Mean characteristics in the spatial map were averages over 43 annual values. The *Right* column compares the statistical distribution of air heat wave (gray) versus riverine heat wave (black) characteristics. Vertical dashed lines represent mean characteristics averaged across all years and sites. Riverine heat waves on average occur at half the frequency and a third intensity of air heat waves, but last twice longer.

heat wave characteristics vary spatially (Fig. 2). Air heat waves have occurred more frequently and lasted longer in the pacific coast, Rockies, and the southeastern region but more intensively in the northern United States. Riverine heat waves have occurred more frequently in the pacific coast, more frequently and intensively in the eastern United States but have no clear spatial pattern for duration. Averaged over all sites (Fig. 2 and *SI Appendix*, Table S2), riverine heat waves generally occur at half the frequency (2.3 versus 4.6 events/y), a third intensity (2.6 versus 7.7 °C/event), but almost double the duration (7.2 versus 4.0 d/event) of air heat waves.

### Riverine heat waves Rise More Rapidly Than Air heat waves.

**Fig. 3.**
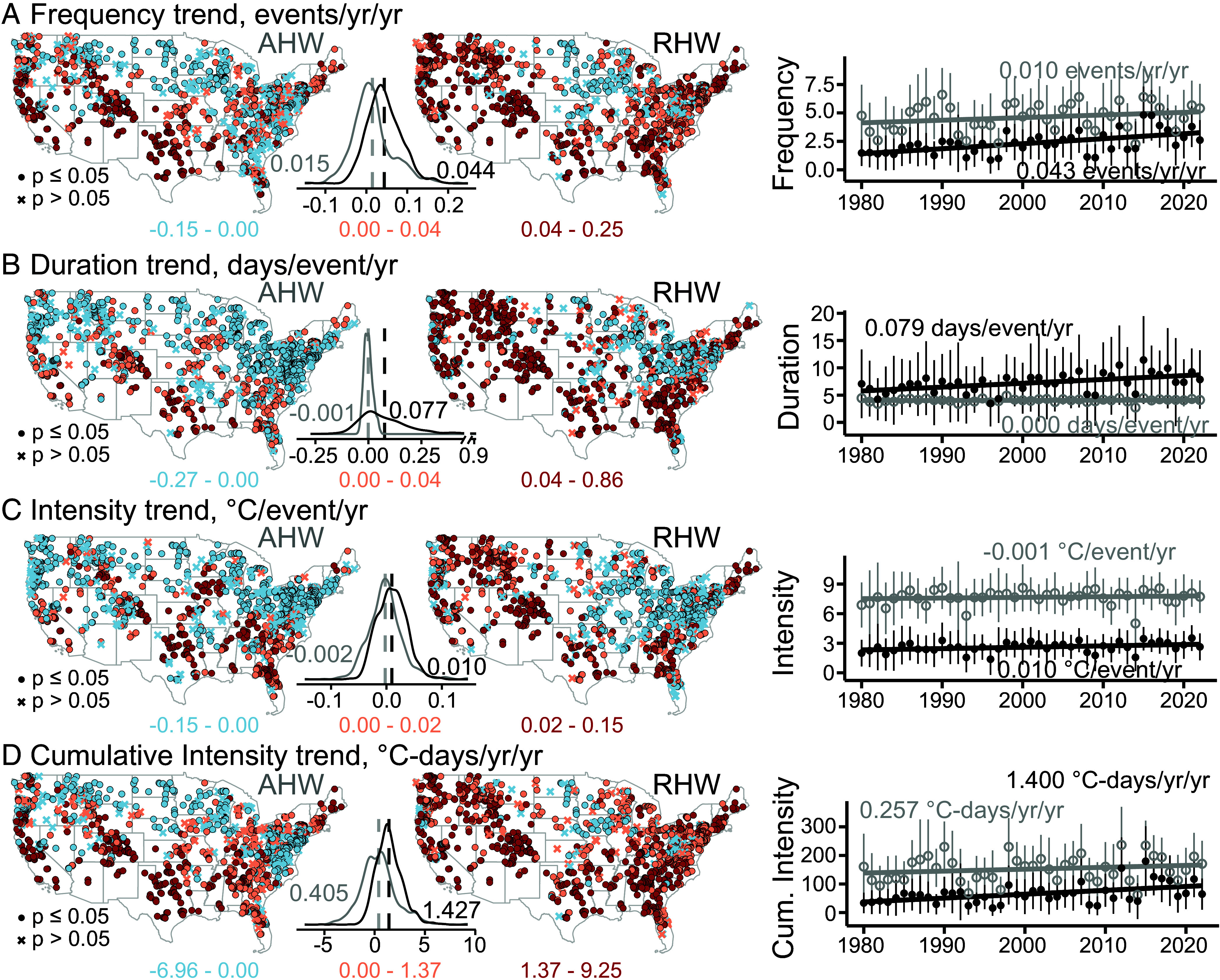
Spatial and statistical distributions of temporal trends of air heat waves (AHW) and riverine heat waves (RHW) characteristics (1980–2022). Trends in annual (*A*) frequency; (*B*) duration; (*C*) intensity; and (*D*) cumulative intensity in the 1,276 best-performing sites. The subplots between maps compare statistical distributions of AHW and RHW trends; the vertical dashed lines are mean trends across all sites. The right column shows trends of annual characteristics averaged across all sites (circles, AHW and RHW in gray and black, respectively), with one SDas error bars. Riverine heat waves have increased more rapidly in all characteristics than air heat waves.

heat waves have generally become more frequent, intense, and longer. Riverine heat waves have increased in all characteristics in most of the United States, declining only in small regions of the midwestern and northern US (Fig. 3 mid column). On average, riverine heat wave frequency has increased, remained relatively constant (insignificant), and declined in about 80%, 12%, and 8% of sites, respectively (Fig. 3 and *SI Appendix*, Fig. S4 and Table S3). It has increased most rapidly in the Rockies and pacific coast but declined rapidly in the intensively cultivated midwestern regions. In comparison, air heat wave frequency has increased in all characteristics across large swaths of the southern US (red) but declined in a much larger area in northern US (blue, Fig. 3), with 45, 27, and 28% of sites showing increasing, insignificant, and declining trends, respectively.

Riverine heat wave duration has become significantly longer (*p* ≤ 0.05) in about 62% of sites, whereas intensity and cumulative intensity have increased in 54 and 81% of the sites, respectively (Fig. 3). Air heat wave duration (Fig. 3*B*) has increased most rapidly in the southern and the southwest region but declined in the Great Lakes region and the midwestern region. Air heat waves (Fig. 3*C*) have intensified mostly in the southern Great Plains and southeastern United States, whereas riverine heat waves have intensified mostly in the Rockies. Air and riverine heat wave intensities have declined in 41% and 28% of sites (*p* < 0.05, negative trends), respectively, mostly in the intensively cultivated Midwest and Great Lakes regions.

Riverine heat waves have increased more rapidly than air heat waves in all characteristics in 65 to 76% of the sites. The mean trends of air and riverine heat waves across all sites (Fig. 3 and *SI Appendix*, Table S2) are 0.015 and 0.044 events/year/year in frequency, -0.001 and 0.077 d/event/year in duration, −0.002 and 0.010 °C/event/year in intensity, and 0.405 and 1.427 °C-d/y/y in cumulative intensity, respectively. In other words, air heat waves have become more frequent but have remained relatively constant on average in duration and intensity, likely due to the large number of heavy agriculture sites experiencing declining trends. Based on the means of statistical distributions (Fig. 3 between spatial maps), riverine heat waves have increased at double to quadruple rates compared to air heat waves. These rates amount to an additional 1.8 events/year in frequency, 3.4 d/event in duration, and 0.43 °C/event in intensity in 2022 compared to 1980.

### Distinct Riverine heat wave Characteristics Under Different Land Uses.

**Fig. 4.**
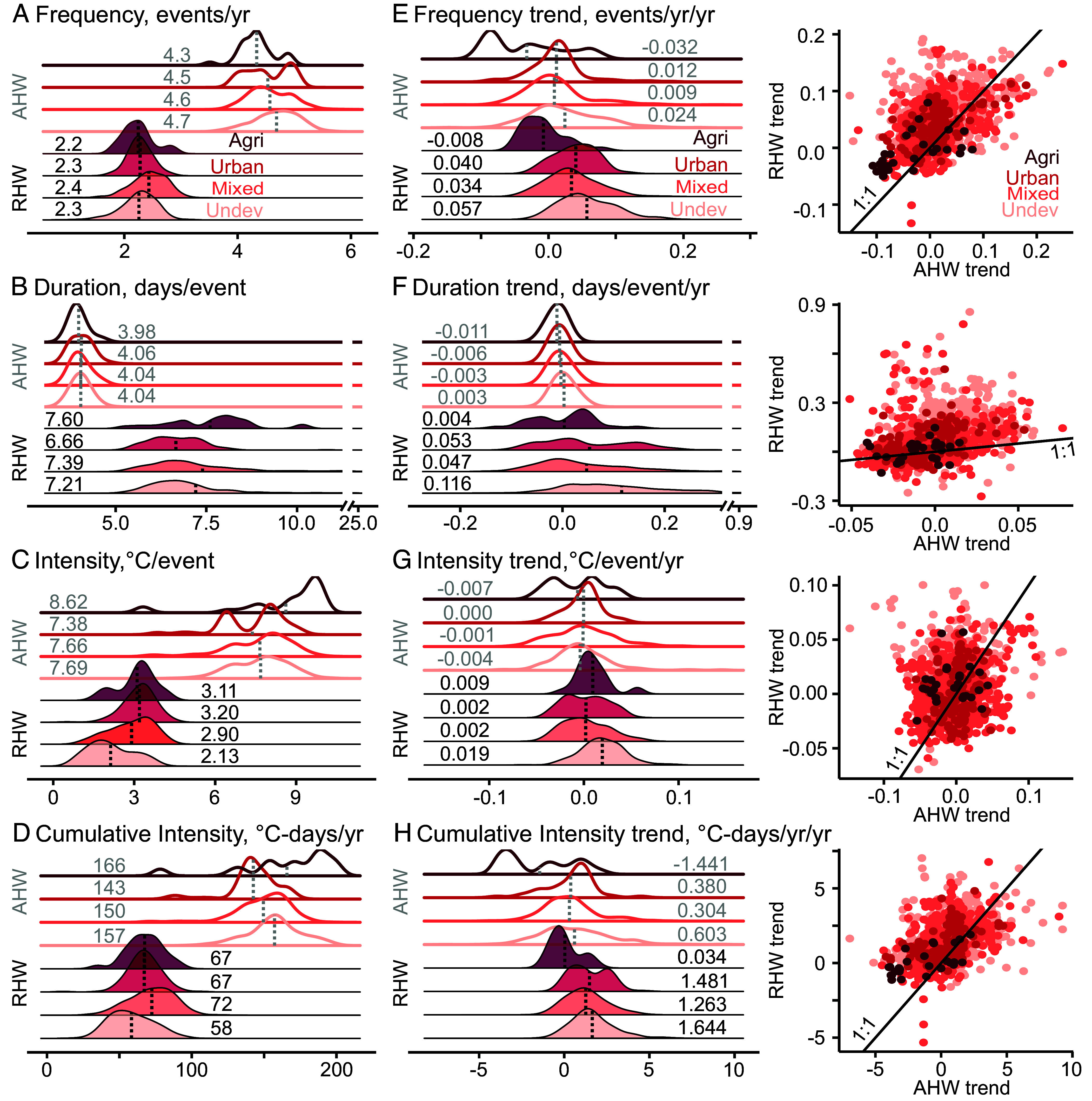
Characteristics (*A*−*D*) and temporal trends (*E*−*H*) of air heat wave (*Top*) and riverine heat wave (*Bottom*) under different land uses. Vertical dashed lines in density plots represent average characteristics or trends. Right column: intercomparison of RHW versus AHW trends in different land uses, with solid lines representing 1:1 relationship. In all event characteristics, riverine heat waves have increased more rapidly than air heat waves. Mean riverine heat waves characteristics and their trends are summarized in *SI Appendix*, Table S2.

The 1,276 sites include 552 undeveloped (43%), 552 mixed (43%), 145 urban (11%), and 27 agricultural (2%) sites based on the classification by the United States Geological Survey (USGS) (*SI Appendix*, Tables S2 and S4 and Fig. S1). Undeveloped sites have the most rapidly rising air and riverine heat waves in all characteristics among the four types of land uses (Fig. 4 *E*–*H*), indicating limited buffering capacity despite their capability of shielding off warming effects. In agricultural sites, riverine heat waves are generally longer and more intense but slightly less frequent than under other land uses (Fig. 4), whereas trends of air heat waves have declined or only slightly increased. In urban sites, riverine heat waves are more intense but shorter than mixed and undeveloped sites. Mixed sites generally lie between undeveloped and agricultural sites; riverine heat waves are longer, more frequent, and more intense compared to undeveloped sites.

Overall, riverine heat waves have increased more rapidly in frequency and cumulative intensity in urban sites, and more rapidly in duration and intensity in undeveloped sites compared to air heat waves. Riverine heat wave frequency has increased more rapidly than air heat waves in 74% of the sites, and in the order of urban (77%) > undeveloped (74%) ≅ mixed (74%) > agricultural (67%). Their duration has increased more rapidly in 75% of the sites, in the order of undeveloped (88%) > urban (73%) > mixed (64%) > agricultural (63%). Their intensity has increased more rapidly in 66% of the sites, with undeveloped (80%) > agricultural (70%) > urban (55%) > mixed (53%). Annual cumulative intensity has increased faster in 76% of the sites, with urban (80%) > mixed (78%) > undeveloped (74%) > agricultural (74%).

### Riverine heat wave Trends Driven Predominantly by Climate-induced Temperature and Water Changes, Followed by Dams and Agriculture Activities.

**Fig. 5.**
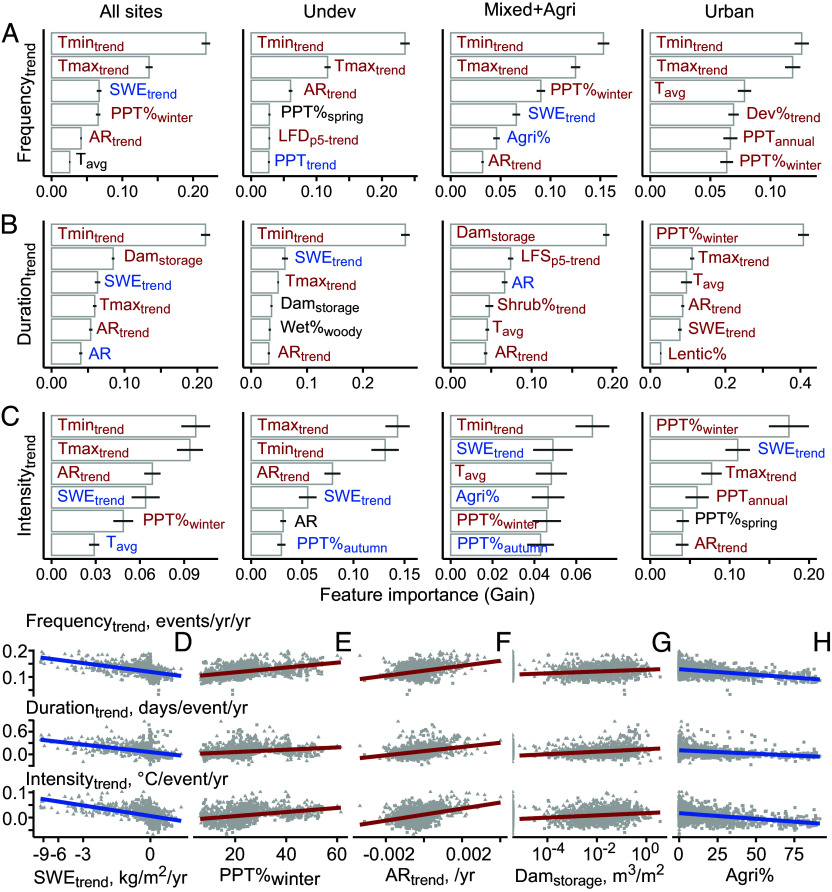
Top influential drivers of riverine heat wave trends for annual (*A*) frequency; (*B*) duration; and (*C*) intensity in all sites (first *Left* column) and under different land uses (second to fourth columns). Each horizontal bar and its error bar indicate mean gain and one SD of 2000 BRT runs, respectively. Red and blue colors indicate significant positive and negative spearman correlation, respectively, with heat wave trends; black represents insignificant correlation (*p* > 0.05). *Bottom* row: relationships between riverine heat wave trends and (*D*) trends in snow water equivalent (SWE_trend_); (*E*) winter precipitation percent (PPT%_winter_); (*F*) trend in amplitude ratio (AR_trend_); (*G*) dam water storage (Dam_storage_); and (*H*) percent agricultural cover (Agri%). Subscript “trend” represents trends for corresponding variables. Subscript “annual”, “seasonal”, “winter” describe annual mean, seasonality, and winter characteristics for corresponding variables. For example, Tmin_trend_ and Tmax_trend_ are trends in maximum and minimum AT, respectively; T_avg_ is average AT; Lentic% refers to percent cover of water bodies; Dev%, Agri%, Forest%, and Shrub% are the percent of developed, agricultural, forest, and shrub cover, respectively; Lines indicate linear fit; colors indicate spearman correlation. Top 10 influential drivers are detailed in *SI Appendix*, Fig. S4.

Among 52 attributes including both static land attributes and trends in climate, hydrology, and land use characteristics (*SI Appendix*, Tables S1, S5), we ranked influential drivers of riverine heat wave trends using the BRT model (Fig. 5, Methods). Climate drivers, especially trends of minimum and maximum AT, consistently rank as the top drivers and correlates positively (red) with heat wave trends. Immediately following are other climate characteristics including winter precipitation percentage (PPT%_winter_) and trends of snow water equivalent (SWE_trend_), often correlating negatively (blue) with rising heat waves. Groundwater contribution, quantified as the amplitude ratio of water and AT (AR and AR_trend_) (30, 40), also emerges in the top five attributes, underscoring the essential role of more groundwater in buffering warming climate conditions. Trends in low streamflow (below 5th percentile) characteristics also rank in the top ten (*SI Appendix*, Fig. S4), including the trends in the annual number of low-flow days (LFD_p5-trend_), annual average flow (LFQ_p5-trend_), and annual average flow shortage (LFS_p5-trend_) during low-flow days. Subsurface characteristics such as water table depth and soil properties influence heat wave trends to some extent, although not predominantly.

heat wave trends are additionally shaped by human infrastructure and activities such as dams and agriculture (Fig. 5). In particular, the water storage of dams (Dam_storage_) is the most influential in elongating heat waves. The trends in the frequency and intensity of river heat waves in mixed and agriculture sites decrease with higher percentage of agriculture land (Agri%), not surprising given the cooling trends observed in the Midwest (Fig. 3). Trends of agricultural cover however are not important, as land use has mostly stabilized in the past few decades (*SI Appendix*, Table S5). Interestingly, although urban heat island effects are commonly perceived as important (32, 41), urban riverine heat waves are predominantly driven by changing AT and winter precipitation. The percentage of developed areas (Dev%) and its trend emerge in the top five only for the trends of heat wave frequency.

## Discussion

Riverine water temperature have been monitored for decades and are known to increase in a warming climate (32, 33), yet research on riverine heat waves are still emerging (21). This is in sharp contrast with the extensive documentation of heat waves in air, lakes and oceans (16–19, 42, 43). Here, the reconstruction of continuous daily data in over a thousand sites with diverse climate and land use conditions enables systematic analysis that answers questions on characteristics, patterns, trends, and drivers of riverine heat waves compared with air heat waves. Results here show less frequent and intense but longer riverine heat waves compared with air heat waves, and more rapid increase in all characteristics. The analysis of influential drivers potentially indicates that climate change drives river–air interactions and serves as the primary external drivers of riverine heat waves. In contrast, land structures such as dams and agriculture shaped by human activities serve as the secondary internal drivers that modulate river–land interactions and determine when, where, and to what extent rivers respond to anthropogenic climate change.

### Unique Riverine heat wave Characteristics Compared With Air, Lake, and Marine heat waves.

Contradictory to common perceptions that rivers are cool refuges, riverine heat waves have increased at double to quadruple rates compared with air heat waves in 65 to 76% of the sites. Compared with lake heat waves (16), they are similarly long but less intensive (*SI Appendix*, Text S2 and Fig. S6 and Table S6). Compared with marine heat waves of similar definition (category I) (42), riverine heat waves are less frequent and shorter but more intense; they are increasing slower in frequency and duration but faster in intensity, although large uncertainties exist (*SI Appendix*, Text S2 and Fig. S6 and Table S6). These results highlight unique river responses to thermal disturbances. Unlike large water bodies with higher thermal inertia, rivers respond rapidly to hot air, hydrological variations, and land-use alterations, all of which make them highly vulnerable (40).

### Climate Changes As the Primary External Driver.

Rising WT has been shown to be regulated by both climate (21, 29, 30) and human drivers (32, 33), although it has remained equivocal which one has stronger influence. With more than a thousand sites under diverse climate and land use conditions, the analysis here unequivocally shows that climate change is the direct, primary driver of the trends in riverine heat waves, although we recognize that climate change itself is driven by human activities. This echoes the findings of climate controls on many other water quality variables (12, 44). Trends of minimum AT often show up at the very top (Fig. 5), possibly due to its faster increase compared with maximum AT (45). Trends in precipitation, especially winter precipitation and snow, are also important, echoing the most rapid heat wave increase in high-elevation mountains (Fig. 4). Shrinking snowpacks reduce thermal buffering provided by gradually released cool meltwater, which can exacerbate riverine heat waves (31, 46).

Changing water characteristics, including trends in groundwater contribution and low streamflow, also substantially influence riverine heat waves. Riverine heat wave characteristics increase most rapidly at sites with decreasing groundwater contribution (increasing AR_trend_). Groundwater responds to AT slowly (47) and buffer hot air impacts on WT (48). Rivers with more groundwater contribution therefore are less sensitive to AT changes (30, 40), whereas rivers receiving more water through overland flow and shallow groundwater are more responsive to AT fluctuation. Similarly, low streamflow reduces thermal capacity of running water, elongates water residence times (49), and exacerbate riverine heat waves (50, 51).

### Human Activities as Secondary Driver.

Although secondary to climate drivers, agriculture and dam characteristics substantially influence trends of riverine heat waves. The cooling trends observed in major agricultural regions such as the Midwest (Fig. 3) echo global decline in AT during growing seasons (52, 53), often attributed to crop intensification (increasing productivity) and irrigation (52, 54). Irrigation can have profound impacts on discharge, groundwater contributions, and heat waves (40, 55). Groundwater-sourced irrigation may increase heat waves by lowering water table and its contribution to streamflow (55). River flow diversions for irrigation can reduce streamflow and warm up rivers, whereas irrigation flow returns via subsurface drains can ameliorate river warming (56). The positive correlation between dam storage and riverine heat wave duration (Fig. 5) indicates that large dams tend to elongate riverine heat waves. This contrasts observations in literature that dams can either increase or alleviate river heat waves, depending on dam location, characteristics, and timing and duration of water release from dams (29, 57, 58). For example, increasing heat wave durations has been observed upstream of dams or reservoirs (21). The release of upper warm epilimnion water has been shown to escalate but the release of deeper cool hypolimnetic water can dampen seasonal variations in WT downstream (57). Results here potentially indicate that in dams with large water volume, stagnant standing water leads to continued increase of WT even at depth, such that even water released at the bottom of reservoir still exacerbates riverine heat waves.

### Deep Learning Models for Water Quality.

Given the availability of observation and input data, results here are inevitably biased in time and space. Sites with longer data gaps or shorter historical records have larger uncertainty (*SI Appendix*, Fig. S7). Nonetheless, the analysis here illustrates the strength of LSTM models in integrating spatially fragmented and temporally inconsistent data under diverse conditions, coined “data synergy” in literature (59). Only 57 of the 1,471 sites have data exceeding 70% d coverage (Fig. 1), the recommended minimum data threshold for training traditional individual-site-based linear regression models (60). The trained model performs well even at sites with only 100 data points across four decades. The trained model can be used for spatial fillings to reconstruct WT data at river stations without data and for hindcasting and forecasting with corresponding input data. The analysis here therefore demonstrates the promises of building LSTM models for broader understanding and projection of water quality in time and space. They can additionally be used for variables with much less data, expanding the use beyond WT. In fact, its use in predicting dissolved oxygen (20) and total phosphorus (61) and for places beyond CONUS have already been demonstrated.

### Cascading Impacts of Riverine heat wave Events Call for Global Actions.

The faster increase of riverine heat waves compared with air heat waves is likely occurring worldwide (62), underscoring the need for coordinated data consolidation and for more balanced monitoring networks. In addition, it also indicates more frequent co-occurrence of hot water and low flow conditions, which can lead to compound events of riverine heat waves and droughts (62). These conditions often reduce dissolved oxygen levels and elevate salinity, among many other water quality variables (11). These conditions can create hypoxic conditions and place osmotic stress on aquatic species. Aquatic life, therefore, experiences not only thermal stress but also hydrological and biogeochemical disturbances. These multiple stressors can exacerbate multifaceted threats to aquatic life, potentially leading to large-scale fish mortality events that have only begun to be systematically documented (3–5). River heat waves also jeopardize water quality and global water, food, and energy security (15, 63). As climate change accelerates, incorporating risks of riverine heat waves into climate adaptation strategies and global water governance frameworks will be essential to safeguard freshwater ecosystems and human society.

## Methods

### Data.

#### Sites.

The deep learning model was trained using data from 1,471 USGS river stations (64) that have catchment attributes in the Geospatial Attributes of Gages for Evaluating Streamflow (GAGES II) database (65). They have daily WT measurements between 100 and 15,464 over 1980–2022, with a mean of 3,405 data points, corresponding to 0.6, 98.5, and 21.7% of day coverage, respectively.

#### Time series data.

Daily mean WT (parameter code “00010”, statistic code “00003”, °C) and mean discharge (parameter code “00060”, statistic code “00003”, ft^3^/s) were downloaded using “dataRetrieval” package in October 2023 (66). Meteorological data is remotely sensed data from Daymet (https://daymet.ornl.gov/overview) (67) downloaded using Google Earth Engine (68); it offers more comprehensive spatial and temporal coverage compared to meteorological station data. It includes daily daylight duration (s/d), precipitation (mm), shortwave radiation (W/m^2^), snow water equivalent (kg/m^2^), maximum and minimum AT (°C), and water vapor pressure (Pa) (67). Data preprocessing is described in *SI Appendix*, Text S3.

#### Static catchment attributes.

Thirty-three attributes from the GAGES II database were used for LSTM model training. They include, for example, climate, hydrology, topography, soil, stream network, and land use attributes (range and median in *SI Appendix*, Table S1). Sites vary in drainage area (1.62 to 49,802 km^2^), mean annual AT (−1.94 to 22.64 °C), mean annual precipitation (20.80 to 416 cm/y), mean elevation (6.4 to 3,600 m), and other characteristics (statistical distributions in *SI Appendix*, Fig. S8). Attribute selection (*SI Appendix*, Text S4 and Fig. S9*A*) and their Pearson and Spearman correlation coefficients are detailed in (*SI Appendix*, Figs. S10 and S11).

### Model.

#### Deep learning model.

The LSTM model is a type of recurrent neural network that overcomes the vanishing gradient problem in traditional recurrent neural networks (36) (*SI Appendix*, Text S5). LSTMs can learn complex temporal dependencies across different sites, making them more efficient than traditional models in modeling time series data (23). One “global” LSTM model was trained using data from all sites to predict daily WT and daily discharge using daily meteorological forcings and static attributes (*SI Appendix*, Fig. S12) with code adapted from hydroDL repository (69). RMSE with equal weight to both WT and discharge was used as loss function. We used discharge as a target instead of input data because discharge data are less available compared to remotely sensed weather data and can restrict the use of the trained model to sites with discharge data.

#### Training versus testing data splitting.

To ensure an even distribution of training and testing data, the time period with WT data was split for each site such that the earlier period with 75% of data serve as the training period, whereas the later period with the rest 25% data serve as the testing period. This flexible splitting has been used previously to ensure maximum spatial and temporal coverage across sites (20). Training and testing periods, therefore, differ for each site due to varying spatiotemporal data coverage. The start of the training period was set as the first date with discharge data, if it was earlier than the date with the first WT data.

#### Hyperparameters tuning.

Hyperparameters were adjusted in sequence, yielding optimum hidden size or number of neurons of 256, sequence length of 365 d, and drop rate of 0.3 that gave lowest median test RMSE values (*SI Appendix*, Fig. S9 *B*–*D*). To account for inherent randomness, the calibrated model was then run with five different seeds, the mean of which was used for further analysis.

#### Testing effects of data availability.

Data coverage is essential for training robust models. LSTM is known for its capability of leveraging diverse datasets from many sites (59, 70). To train one single model for the largest number of sites possible, we tested two versions: LSTM_853_ model that uses 853 sites with at least 1,825 data points in each site (~12% coverage) and LSTM_1471_ that uses 1,471 sites with at least 100 data points in each site. The trained LSTM_853_ had similarly good performance as the trained LSTM_1471_ in the 853 sites (*SI Appendix*, Fig. S13). However, when LSTM_853_ was used to predict WT in the 618 sites in LSTM_1471_ but not in LSTM_853_, the median NSE dropped to about 0.71 compared to 0.99 in LSTM_1471_, and 33% of the sites had NSE lower than 0.5. To maximize the number of sites with best model performance, we used LSTM_1471_ with 100 data point threshold, a threshold that has been used before for training LSTM for dissolved oxygen (20). Increasing the threshold to 500 and 1,000 would have reduced the number of sites to 1,322 and 1,076, respectively.

To further compare model performance at sites with different data availability, we selected groups of 25 sites having data points of 1%, 10%, 25%, 50%, 75%, and 88% (not enough sites with ~100%) data coverage in 1980–2022. Model performance does increase with more data availability (*SI Appendix*, Fig. S7). However, even among sites with only 1% data coverage, median NSE and KGE values are above 0.5, and quickly increase to about 0.9 at 2.5% data coverage.

### Definition, Characteristics, and Trends of heat wave Events.

#### Event definition and characteristics.

Riverine and air heat waves are defined as events when their respective temperature exceeds their corresponding local, seasonally varying 90th percentile threshold for at least 5 and 3 d, respectively (Fig. 1*A*) (17, 71). If two riverine heat wave events (but not air heat waves) are separated by 1 or 2 d below the threshold temperature, they are considered as one single event. Event duration is the number of days over which the temperature exceeds its threshold. Mean event intensity is the mean difference between daily temperature and its historical daily mean during an event, instead of maximum event intensity defined as peak temperature change. The 90^th^ percentile threshold varies for each Julian day and was calculated using temperature data over 43 y (1980–2022), thereby meeting the suggested guideline of using a minimum of 30-y long data records.

Air heat wave characteristics were calculated from remotely sensed daily maximum AT (median over drainage area) from Daymet (67). Riverine heat wave characteristics were calculated using modeled daily mean WT. All characteristics were calculated using the “heat waveR” package (72). Detailed equations are in *SI Appendix*, Text S6. While we used the commonly used 90th percentile threshold (17), different thresholds can lead to different quantification of event characteristics (*SI Appendix*, Text S7 and Fig. S14 and Table S7), although major conclusions remain the same.

We quantified four annual heat wave characteristics—frequency, duration, intensity, and cumulative intensity—for each year in 1980–2022. Annual frequency is the total number of events in a year; annual duration (days/event) is the average of event duration; and annual intensity (°C/event) is the average of mean event intensity of all events in a year. Annual cumulative intensity (days-°C/y) is the sum of the products of event duration and mean event intensity of all events in a year. Annual frequency, duration, and intensity are zero in years with no heat wave events.

*Trends:*The trends in heat wave characteristics were quantified as modified Sen’s slope using Mann–Kendall test with trend free prewhitening over a 10-y running window (73, 74) using the R package “modifiedmk” (75). Running window helps detect trends that are otherwise masked by interannual variations. In years without events, the annual values are zero. Over 70% and 80% of sites showed significant trends in air and riverine heat wave characteristics (p ≤ 0.05). Sites with insignificant trends typically have close to zero values (*SI Appendix*, Fig. S4). Therefore, trends in all 1,276 sites were used irrespective of their significance. We additionally compared riverine heat wave characteristics and trends calculated from modeled and observed WT at 53 sites with at least 75% of data coverage (30 y), the recommended threshold for heat wave analysis. The values from the measured and modeled data are mostly at the vicinity of the 1:1 line, have similar ranges and are highly correlated (*SI Appendix*, Table S8 and Fig. S15), indicating that trends from modeled data are representative of those from real data.

### Influential Drivers of Riverine heat wave Trends.

We used the R package “XGBoost” (76) for BRT model to identify influential drivers of riverine heat wave trends. BRT fits several classification and regression trees using stochastic gradient descent method, with each successive tree focusing more on improving predictions for poorly predicted instances (37). We used 52 attributes as candidates, including 33 static attributes from GAGES II database (*SI Appendix*, Table S1) and 19 additional trend attributes (*SI Appendix*, Table S5). More details are in *SI Appendix*, Texts S8 and S9.

## Supplementary Material

Appendix 01 (PDF)

## Data Availability

Data are from public databases—Daymet (67), USGS Water Data for the Nation (64), and GAGES II (65). The modeled daily riverine water temperature in the 1,471 sites over 1980–2022 can be downloaded in Zenodo (77). The LSTM model code are in Zenodo (78). The code used to extract heat wave characteristics are in Zenodo (79). Modeled river water temperature data used in the paper “Riverine heat waves on the rise, outpacing air heat waves” data have been deposited in Zenodo (https://doi.org/10.5281/zenodo.13718195).
