# Supplementary material for: Riverine heat waves on the rise, outpacing air heat waves
Source: Proc Natl Acad Sci U S A. 2025 Sep 22;122(39):e2503160122. doi: 10.1073/pnas.2503160122 (PMC12501193; doi:10.1073/pnas.2503160122)
Supplement: Supplementary file 1 — Appendix 01 (PDF) [file pnas.2503160122.sapp.pdf]

PNAS

Supporting Information for

Riverine heat waves on the rise, outpacing air heat waves

Kayalvizhi Sadayappan[1], Li Li[1*]

[1]Department of Civil and Environmental Engineering, The Pennsylvania State University; University Park, 16802, USA.

[*]Corresponding author. Email: lili@engr.psu.edu

This PDF file includes:

Supporting text S1 to S9

Figures S1 to S15

Tables S1 to S8

SI References

Supporting Text

S1: Quantifying thermal stress and critical thermal stress days

To quantify the increase in hot water days that could cause thermal stress for aquatic life, we chose two threshold temperatures, 15°C and 20°C and defined days exceeding these temperatures as thermal and critical thermal stress days, respectively. We grouped states into six regions: NorthEast – Maine, New Hampshire, Vermont, Massachusetts, Connecticut, New York, New Jersey, Pennsylvania, Washington D.C., Rhode Islands, Delaware, Maryland; Pacific Northwest – Washington, Oregon, Idaho, California; Rocky Mountain region – Wyoming, Montana, Colorado, Utah; MidWest – Wisconsin, Illinois, Michigan, Ohio, Indiana, North Dakota, South Dakota, Minnesota, Nebraska, Iowa, Missouri, Kansas; Appalachian – North Carolina, Tennessee, Virginia, West Virginia, Kentucky; Southern – Alabama, Arizona, Arkansas, Florida, Georgia, Louisiana, Maryland, Mississippi, Nevada, New Mexico, Oklahoma, South Carolina and Texas. For each region, the number of annual days exceeding the thresholds were averaged across all sites located in them. The trends were quantified as modified Sen's slope over time series of annual thermal and critical thermal stress days using Mann-Kendall test with trend free pre-whitening over a 10-year running window (1, 2) using the R package "modifiedmk"(3). Please refer to Fig.1 and Fig. S3.

S2: Comparison of riverine heat waves with air, marine and lake heat waves

Fig S5 and Table S6 compares different types of heat waves – air, marine, lake and riverine. Heat waves can be classified into several categories. While this study does not differentiate between categories, the majority of the riverine heat waves belong to Category I according to marine heat wave standards (4). We therefore only considered category I statistics for marine heat wave studies that differentiate heat wave categories. The figure shows that riverine heat waves occur on average at a frequency of 2.3 events/year and an intensity of 2.6 °C /event, and last 7.2 days/event. Marine heat waves (Category I) are slightly more frequent, occurring on average at 2.7 events/year, and last significantly longer at 14 days/event (5). Marine heat waves however have lower intensity of 1.3 °C/event (5). Lake HWs have similar duration (7.7 days/event) but higher intensity (3.7 °C/event) than riverine heat waves (6).

In terms of trends, riverine heat waves generally have slower increasing trends in frequency at 0.044 events/year/year compared with marine heat waves of (0.09 events/year/year) (5). Another marine heat wave study has also reported 0.045 events/year/year (7). Riverine heat wave duration has increased less rapidly at 0.077 days/event/year compared with marine heat waves ranging from 0.13 to 0.23 days/event/year (5, 7, 8). Riverine heat waves have a mean intensity trend of 0.010 °C/event/year in this work compared to 0.0085 (7), 0.0005 (8), and -0.0009 (5) °C/event/year for marine heat waves. We could not find equivalent trends in lake heat wave characteristics in literature. In other words, riverine heat waves are generally less frequent and shorter but more intense than marine heat waves, both in terms of characteristics and their trends.

S3: Data preprocessing

Water temperature measurements below -5°C were eliminated, considering that even seawater with high salt content typically freezes at such low temperatures. Sites with hot springs and hence abnormally hot water temperatures were also excluded. Similarly, water temperature values above its corresponding maximum air temperature over 1980-2022 were removed. River discharge values below zero were considered missing values. Daymet data cover 365 days each year. For leap years, data on 29th February is included but data for 31st December is excluded. The same procedure was used to prepare daily discharge and water temperature records. Input data were transformed and standardized

using the package "bestNormalize" (9) because deep learning models are more stable and converge better when input data is normalized.

S4: Selection of relevant site attributes

The model was initially built with 441 constant attributes from GAGES II. We combined Boosted Regression Tree (BRT), a machine learning approach, and correlation – both Pearson and Spearman – analysis to identify site characteristics that best explained trends in riverine heat wave characteristics (10, 11) and included them in final model with 33 selected attributes (Table S1).

Correlations between trends in riverine heat wave characteristics and various site attributes were first evaluated to identify potential candidates for BRT model. Both Pearson and Spearman correlation coefficients quantify the direction and magnitude of relationship between two variables and vary between -1 and 1. Negative coefficient indicates an inverse relationship and vice versa. Pearson correlation quantifies the linear relationship between two variables. Spearman correlation, however, quantifies relationship between ranks of two variables and therefore can identify non-linear but monotonic relationships. While both correlations were calculated, spearman correlation was given more importance as it can capture non-linear monotonic relationships as well.

Among the attributes, we chose the ones that are relatively independent with low correlation. However, in some cases, despite high correlation between two characteristics, they were retained as representing important characteristics. For example, mean site elevation was retained despite its high correlation with mean annual air temperature and percent developed area. Dam, catchment area and stream network related characteristics were retained based on literature, despite their weak correlation with the trends in RHW characteristics (12, 13). The ability of BRT model to detect complex nonlinear relationships helped identify dam storage as an important site attribute, which the correlation analysis was unable to uncover. The most important constant site characteristics as identified by BRT model were included as catchment attributes in the final deep learning model.

The final model showed slightly better performance with lower median test RMSE than the model using all 441 attributes (Fig. S8A). We therefore used the model with 33 attributes. Pearson and spearman correlation coefficients among these attributes are in Fig. S9-10.

S5: Long Short-Term Memory model structure

The Long Short-Term Memory (LSTM) model is a type of recurrent neural network that uses gates, namely input gate, forget gate and output gate, to selectively retain important information while forgetting inconsequential information (14). It can therefore better learn and remember long-term dependencies in sequential data like time-series and can learn non-linear and complex patterns from data.

Input transformation: $x_t = \mathrm{Re}\,LU(W_x x(x_t^0) + b_x x)$

Forget gate: $f(t) = \sigma(W_{fx} D(x_t) + W_{fh} D(h_{t-1}) + b_f)$

Input gate: $i(t) = \sigma(W_{ix} D(x_t) + W_{ih} D(h_{t-1}) + b_i)$

Input node: $g(t) = \tanh(W_{gx} D(x_t) + W_{gh} D(h_{t-1}) + b_g)$

Cell state: $c_t = D(g(t)) \otimes i(t) + c_{t-1} \otimes f(t)$

Output gate: $o(t) = \sigma(W_{ox} D(x_t) + W_{oh} D(h_{t-1}) + b_o)$

Hidden state: $h_t = \tanh(c_{t-1}) \otimes o(t)$

Output: $y_t = W_{hy} h_y + b_y$

where x_t^0 is the raw input at time step t, ReLU is the rectified linear unit activation function, σ is the activation function, $\otimes$ denotes point wise multiplication, x_t is the transformed input at time step t, y_t is the output at time t, $f(t)$ is the forget gate, $i(t)$ is the input gate, $o(t)$ is the output gate, h is the hidden state, c is the cell state, W denotes the network weights, b denotes the constant bias parameters and D denotes the dropout mask. Please refer to Fig. S11 for model structure.

S6: Calculation of riverine heat wave characteristics

We calculated the *threshold mean climatological temperature* $T_m(j)$ (°C) as follows (15):

$$T_m(j) = \sum_{y=y_s}^{y_e} \sum_{d=j-w}^{j+w} \frac{T(y,d)}{(2w+1)(y_e - y_s + 1)},$$

where y_s and y_e are the start and end of climatological base period, j is the Julian day of the year, w is the half window length (7 and 5 days for air and riverine heat waves, respectively), $T\ (y,d)$ is the temperature (°C) on day d of year y (daily maximum air and mean water temperatures). The *seasonally varying 90th percentile threshold temperature* (°C) $T_{90}(j)$ was calculated as follows (15):

$$T_{90}(j) = P_{90}\{T(y,d)|y_s \le y \le y_e, j-w \le d \le j+w\},$$

where P_{90} is the 90th percentile over the distribution $T(y,d)|y_s \le y \le y_e, j-w \le d \le j+w$. $T_{90}(j)$ and $T_m(j)$ were calculated for each of the Julian day using data over 1980 to 2022. They differ for different Julian days but remain constant for same Julian day in different years. *Mean event intensity* was calculated as the arithmetic mean of difference between temperature and climatological ($T_{m,}$ historical 50th percentile) temperature over all days during that event. *Mean event intensity* was quantified as:

$$Mean\ event\ intensity = \frac{\sum_{t=t_s}^{t_e} T(t) - T_m(j(t))}{t_e - t_s + 1},$$

where t_s and t_e are the start and end dates of heat wave event, $T(t)$ is the air or water temperature (°C) on t, $T_m(j(t))$ is the mean climatological temperature (°C) of Julian day j corresponding to date t. *Annual cumulative intensity* is the sum of the products of event duration and mean event intensity of all events in a year (15), calculated as

$$Annual\ cumulative\ intensity = \sum_{i=1}^{n} \left| \int_{t_s}^{t_e} (T(t) - T_m(j(t)))dt \right|_i,$$

where n is the number of events in a year, t_s and t_e are the start and end dates of events, $T(t)$ is the temperature (°C) on date t, $T_m(j(t))$ is the mean climatological temperature (°C) of Julian day j of the year corresponding to date t. Please refer to Fig. 1A for heat wave definition.

S7: Riverine heat wave characteristics under different thresholds

The 90th percentile threshold was used to identify heat wave events following the common practice in marine heat wave studies (15). We admit that using different threshold percentiles can alter

the annual heat wave characteristics and their trends. Fig. S13 shows the distribution of riverine heat wave characteristics and their trends when 85th, 90th and 95th thresholds are used as an example. While their absolute values change as threshold is changed, the characteristics and trends under different thresholds are still highly correlated (0.72-0.99) with each other (Table S7). Therefore, despite the considerable differences in absolute values, we adopted 90th percentile as threshold for our analysis as it is commonly used for identifying riverine and air heat waves.

S8: Calculation of trends in climate, water, and land use features

We calculated a total of 19 attributes including 17 trend attributes for climate, water, and land uses features. These attributes were included in BRT model as potential influential drivers (Table S5).

Trends in climate factors including daily maximum and minimum air temperatures, precipitation, and snow water equivalent (water in snowpack) were estimated based on daily time series of Daymet data. Observed daily discharge time series was gap-filled with LSTM predictions and area normalized (area from GAGES II) to get continuous daily data. Average annual discharge and several low-flow related trends were calculated from this daily data. For each site, low-flow days were identified as days with mean discharge lower than 5th percentile over 1980-2022. We then calculated annual statistics including number of low-flow days, mean daily discharge during low-flow days (daily discharge averaged over all low-flow days in a year), and mean daily water shortage during low-flow days (average of daily difference between discharge and 5th percentile value over all low-flow days in a year). Low-flow related trends were calculated on these annual times series.

Stream water/air amplitude ratio was calculated as the ratio of amplitude of sine wave fitted to predicted daily water temperature (from the deep learning model developed in this study) to amplitude of sine wave fitted to daily average air temperature (mean of daily maximum and minimum air temperature from Daymet) over 1980-2022. Stream water/air amplitude ratio is an indicator of relative contribution of groundwater (16). Lower amplitude ratio means higher relative groundwater contribution as more groundwater contribution dampens the water temperature variations relative to air temperature. Amplitude ratios can be calculated over any time period, though a minimum of a year-long record is recommended. Here the amplitude ratios were calculated for each year in 1980-2022 and trends in the annual amplitude ratio were used as proxy for trend in relative groundwater contribution.

Trends in annual landcover and land use were derived from annual National Land Cover Database developed by Multi-Resolution Land Characteristics Consortium – available for years 1985-2022 (17). These include trends in percent area of forest (sum of classes 41, 42 and 43), developed (sum of classes 21, 22, 23 and 24), water (class 11), barren land (class 31), shrubland (sum of classes 51 and 52), herbaceous (sum of classes 71, 72, 73 and 74), agricultural (sum of classes 81 and 82) and wetlands (sum of classes 90 and 95).

All trends were quantified as Sen's slope using Mann-Kendall test with trend free pre-whitening over a 10-year running window(1, 2) using the R package "modifiedmk". The trends were calculated over daily time series of Daymet and LSTM data and annual time series of landcover data and stream water/air amplitude ratio.

Please refer to the list in Table S5.

S9: Identification of drivers of trends in riverine heat wave characteristics

Attributes for BRT models: Fifty-two attributes were included in BRT model. This includes 33 static attributes from GAGES II database used as input for the LSTM model (Table S1) and 19 additional attributes discussed in S8 and summarized in Table S5.

BRT model fitting: Three BRT models were developed to predict the trend in riverine heat wave frequency, duration and intensity respectively. All attributes were normalized to have a mean of 0 and

standard deviation of 1 before they were used as inputs for BRT (18, 19). The BRT models were calibrated for different parameters including learning rate (0.001, 0.005, 0.01, 0.05, 0.1), maximum depth of tree (3-10 at increment of 1), minimum sum of instance weight in child (8-20 at increment of 1), subsample ratio of data (0.3-1 at increment of 0.1), and subsample ratio of candidates (0.3-1 at increment of 0.1). These parameters were fit in the order presented above. To prevent over-fitting, we used parameters that gave least mean RMSE over held out data in 10-fold cross-validation for 10 different model runs with different seeds (1-10) (18, 19).

Identifying important attributes: With the developed BRT model, we then used gain, the average improvement in model performance when an attribute is included in the model, to rank the importance of attributes for each heat wave characteristic trend (20). As randomness is inherent in machine learning models, we ran each model with different seeds for 2,000 times and used mean gain across 2000 model runs for each attribute to rank the importance of attributes. The ranking of influential drivers can vary with small number of runs but generally converge at about 250 runs. For each trend, BRT models calibrated for all sites were also run individually for sites of different land uses to rank important attributes for particular land uses. Agricultural sites were grouped with mixed land use because there were only 27 agricultural sites.

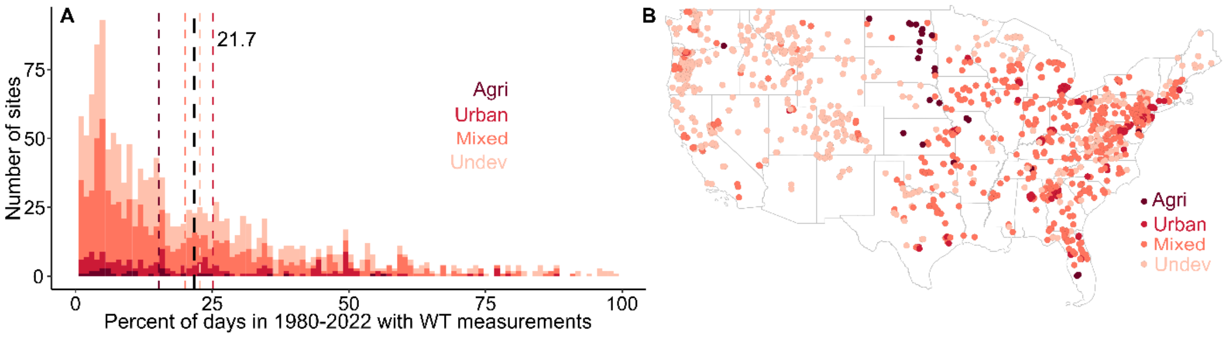

Fig. S1. Data availability and map of sites. (**A**) Distribution of percent days over 1980-2022 with daily mean water temperature measurements (WT, USGS parameter code "00010", statistic code "00003") among 1471 sites used in this study with vertical dashed lines representing mean and color representing different land uses. Black vertical dashed line is the mean over all sites irrespective of land use. Note that the number of sites of different land uses are stacked vertically, adding to the total number of sites in each bin. (**B**) US map showing land use of 1276 sites with best performance. Mixed and urban sites are located mainly in the Eastern US; undeveloped sites are concentrated in the Western US. There are only tens of agricultural sites, located mostly in northern Great Plains.

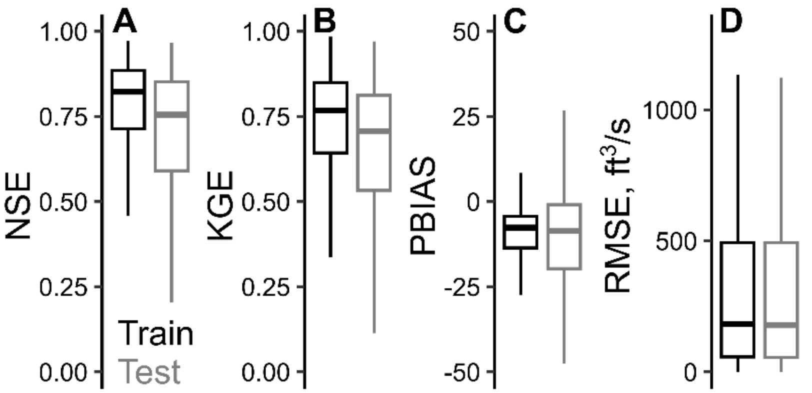

Fig. S2. **Model performance for daily mean discharge predictions.** Summary (25th, 50th and 75th percentiles) of daily scale model performance metrics for mean daily discharge among 1471 sites over training (black) and testing (grey) periods - (**A**) Nash-Sutcliffe Efficiency (NSE); (**B**) Kling-Gupta Efficiency (KGE); (**C**) Percent Bias (PBIAS) and (**D**) Root Mean Square Error (RMSE). NSE and KGE vary form -∞ to 1, with 1 representing perfect performance. Lower RMSE and closer to zero PBIAS values indicate better model performance.

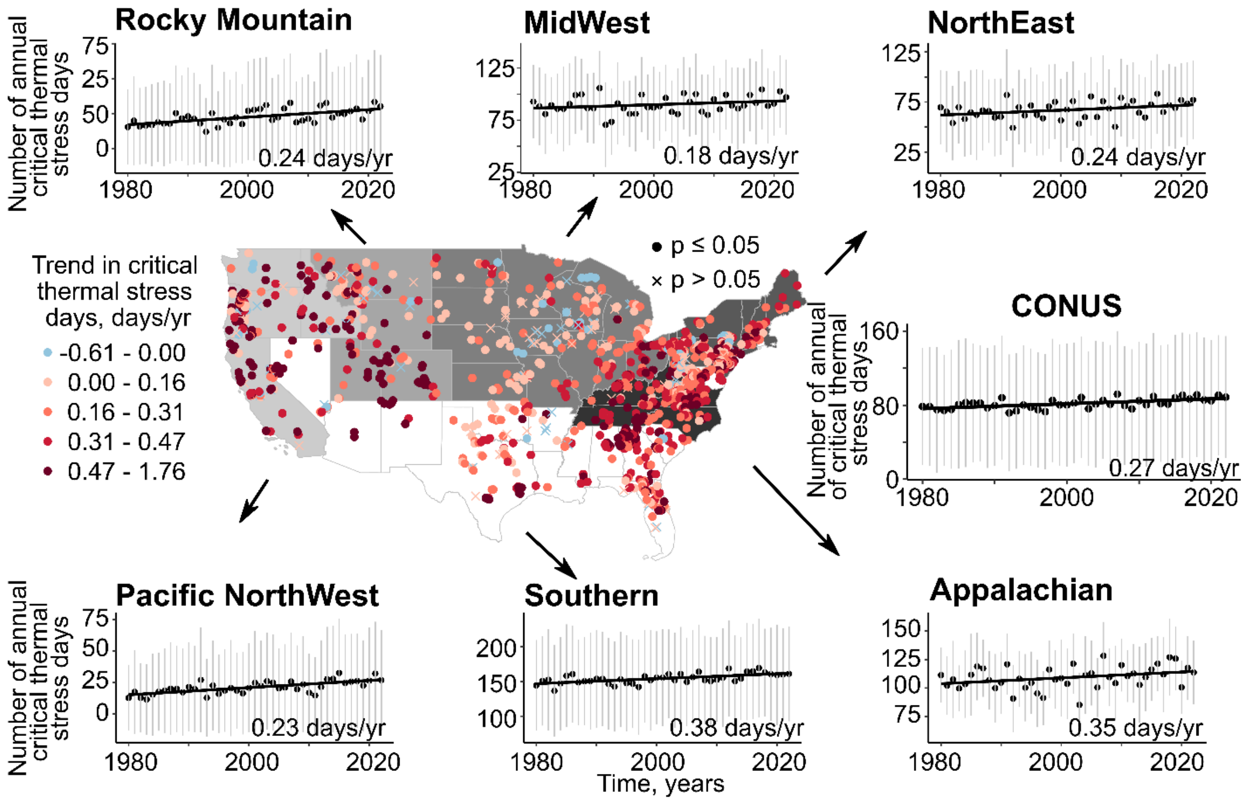

Fig. S3. Trends in annual critical thermal stress days. Central US map shows trends in annual critical thermal stress days (water temperature > 20°C) in 1276 sites over 1980-2022. Side figures are the time series of annual critical thermal stress days (error bars indicate one standard deviation) and their trends in different regions. Regions: NorthEast – Maine, New Hampshire, Vermont, Massachusetts, Connecticut, New York, New Jersey, Pennsylvania, Washington D.C., Rhode Islands, Delaware, Maryland; Pacific Northwest – Washington, Oregon, Idaho, California; Rocky Mountain region – Wyoming, Montana, Colorado, Utah; MidWest – Wisconsin, Illinois, Michigan, Ohio, Indiana, North Dakota, South Dakota, Minnesota, Nebraska, Iowa, Missouri, Kansas; Appalachian – North Carolina, Tennessee, Virginia, West Virginia, Kentucky; Southern – Alabama, Arizona, Arkansas, Florida, Georgia, Louisiana, Maryland, Mississippi, Nevada, New Mexico, Oklahoma, South Carolina and Texas. Only sites with at least 10 annual critical thermal stress days are shown on the US map.

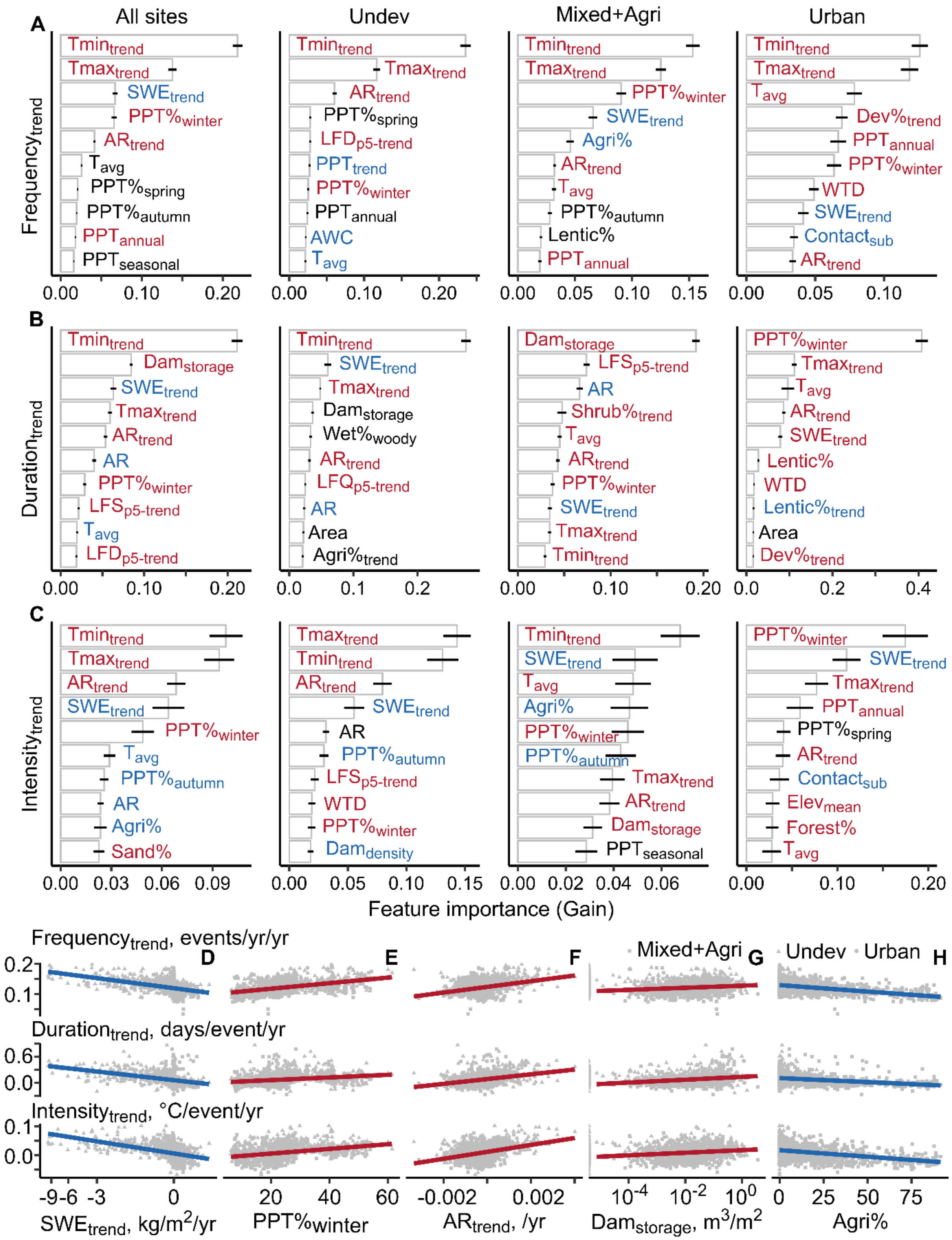

Fig. S4. Site characteristics and trends ranked as top 10 influential drivers of riverine heat wave trends for annual (**A**) frequency; (**B**) duration; and (**C**) intensity in all sites (first column from left) and under different land uses (second to fourth columns). Each horizontal bar and its error bar

indicate the mean gain and one standard deviation of 2000 BRT runs respectively. Red and blue color font indicate significant positive and negative spearman correlation respectively with heat wave trends; black represents insignificant correlation ($p > 0.05$). (**D-H**) Relationships between riverine heat wave trends and (**D**) trends in snow water equivalent (SWE_{trend}); (**E**) winter precipitation percent ($PPT\%_{winter}$); (**F**) trend in amplitude ratio (AR_{trend}); (**G**) dam water storage ($Dam_{storage}$); and (**H**) percent agricultural cover (Agri%). $Tmin_{trend}$ and $Tmax_{trend}$ are trends in maximum and minimum air temperatures (°C/year), respectively; T_{avg} is average air temperature (°C); PPT_{annual} and PPT_{trend} are mean annual and trends in precipitation (cm and mm/year/year), respectively; $PPT\%_{spring}$ and $PPT\%_{autumn}$ are spring and autumn percent precipitation, respectively; $PPT_{seasonal}$ is the seasonality in precipitation with higher values for higher seasonality; $LFD_{p5\text{-}trend}$ is the trend in the number of low-flow days, i.e., days with discharge less than 5th percentile discharge (days/year/year); $LFQ_{p5\text{-}trend}$ and $LFS_{p5\text{-}trend}$ are trends in annual average discharge (mm/day/year) and water shortage (difference in discharge and 5th percentile, mm/day/year) during low-flow days; $Dam_{density}$ is the density of dams (number/100 km^2); AR is the amplitude ratio; Lentic% and $Lentic\%_{trend}$ refer to percent cover of water bodies (%) and its trend (%/year); Dev% and $Dev\%_{trend}$ refer to percent cover of developed area (%) and its trend (%/year); $Agri\%_{trend}$ is the trend in agricultural cover (%/year); Forest% is the percent forest cover (%); $Wet\%_{woody}$ is the percent woody wetland area (%); $Elev_{mean}$ is the mean catchment elevation (m); Area is catchment drainage area (km^2); AWC is the available soil water capacity (depth of water per unit depth of soil); WTD is the depth to water table (meter); $Contact_{sub}$ refers to the subsurface flow contact time (days); Sand% is the percent soil sand content. Lines indicate linear fit; colors indicate spearman correlation.

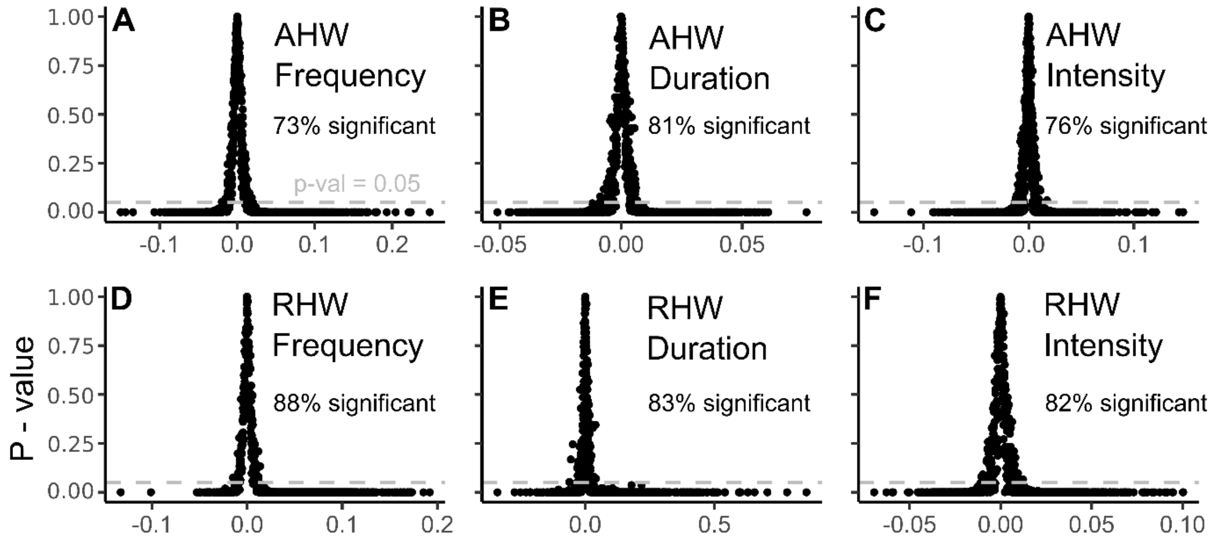

Fig. S5. **P-value against trend** in annual (**A**) air heat wave frequency, number/year/year; (**B**) air heat wave duration, days/event/year; (**C**) air heat wave intensity, °C/event/year; (**D**) riverine heat wave frequency, number/year/year; (**E**) riverine heat wave duration, days/event/year; (**F**) riverine heat wave intensity, °C/event/year for 1276 sites. Over 70% and 80% of sites have significant trends ($p < 0.05$) in air and riverine heat wave characteristics respectively. Insignificant trends are mostly exhibited by sites with trends of magnitude close to zero.

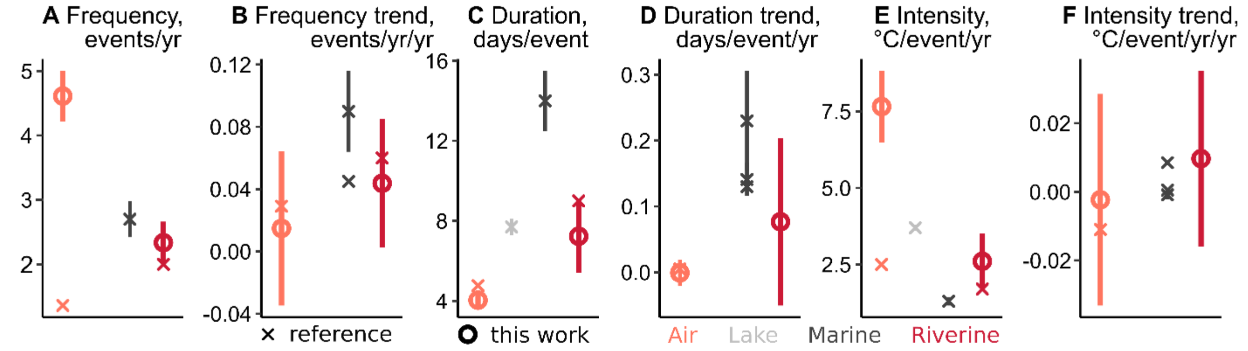

Fig. S6. Comparison of heat wave characteristics and trends from this work and literature. Comparison of annual (**A**) frequency; (**B**) frequency trend; (**C**) duration; (**D**) duration trend; (**E**) intensity; and (**F**) intensity trend for air, lake, marine and riverine heat waves from this work (circle) and literature (cross) (5-8, 21, 22). Symbols represent mean value while error bars represent either one standard error or standard of deviation. Please refer to Table S6 for specific numbers and corresponding references.

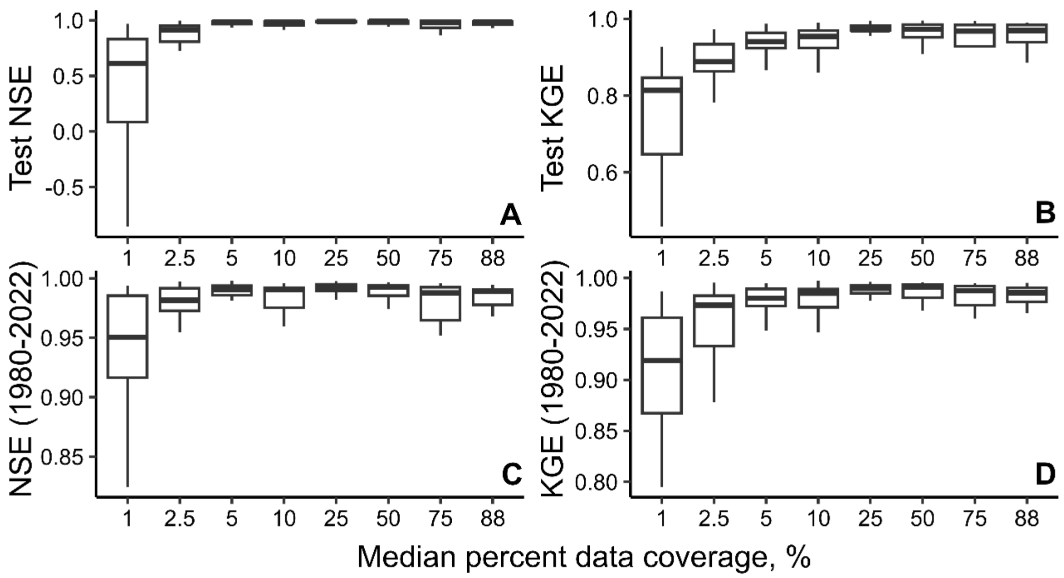

Fig. S7. **Model performance in sites of different data availability** (varying from 1% to 88% data coverage). (**A)** NSE; and (**B**) KGE values during testing periods; (**C**) NSE; and (**D**) KGE over 1980 – 2022. Each boxplot represents 25 sites and its label is their median percent data coverage.

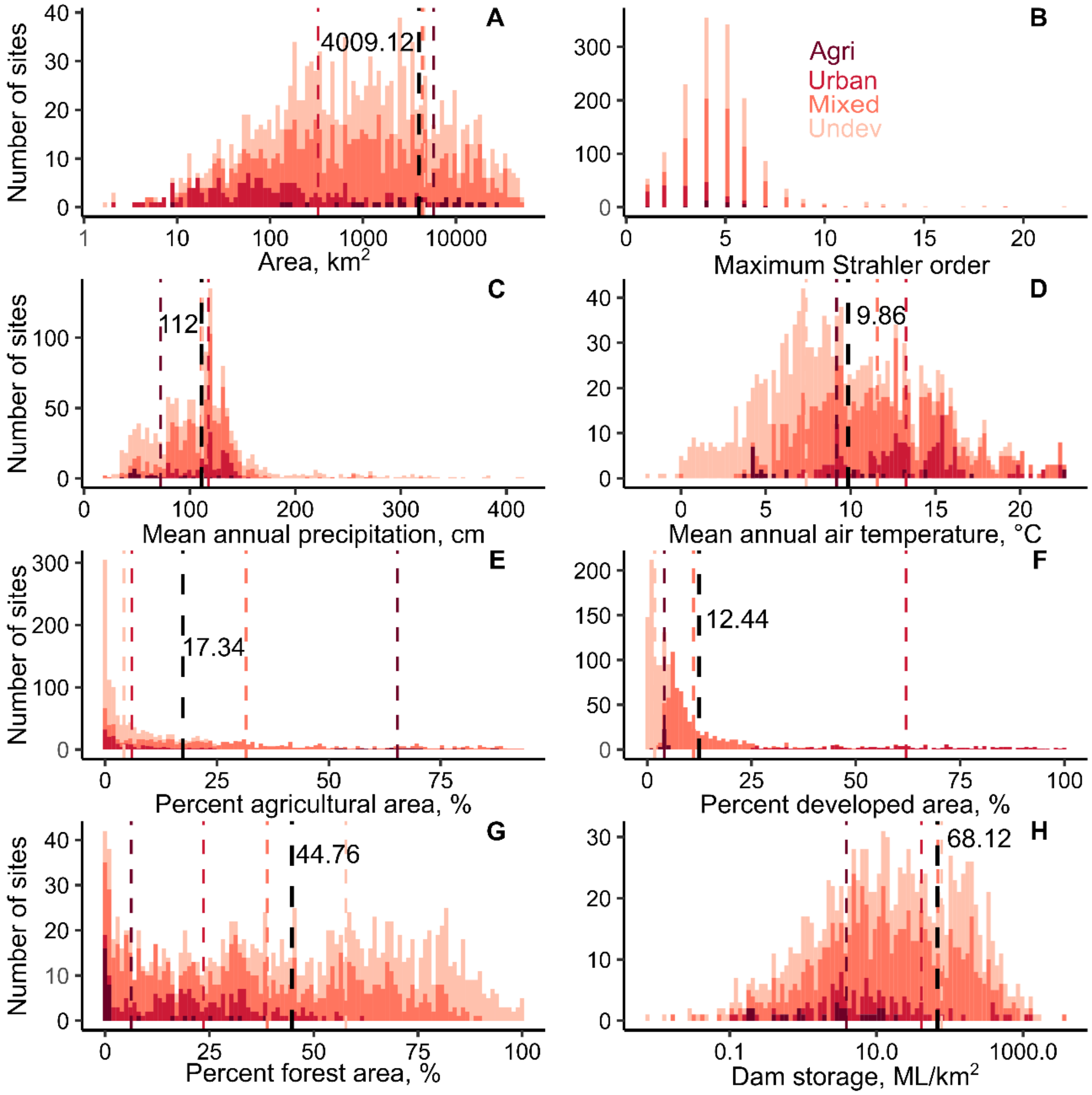

Fig. S8. **Distribution of catchment characteristics**: (**A**) Catchment drainage area (km^2), (**B**) Maximum Strahler order of the stream, (**C**) Mean annual precipitation (cm), (**D**) Mean annual air temperature (°C), (**E**) Percent agricultural area (%), (**F**) Percent developed area (%), (**G**) Percent forested area (%) and (**H**) Dam storage (ML/km^2) among the 1471 sites with vertical dashed lines representing mean and color representing different land uses. Black vertical dashed line is the mean over all sites irrespective of land use. Note that the number of sites of different land uses are stacked vertically, adding to total number of sites in each bin.

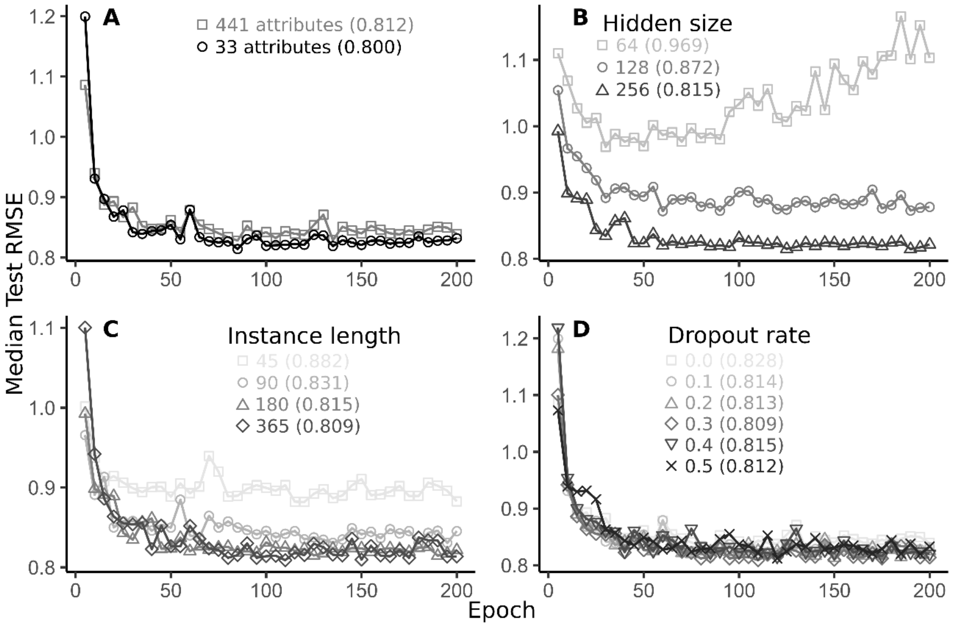

Fig. S9. **Median test Root Mean Square Error (RMSE) for model run** with different (**A**) site attributes; and hyperparameters of (**B**) hidden size; (**C**) instance length; and (**D**) dropout rate.

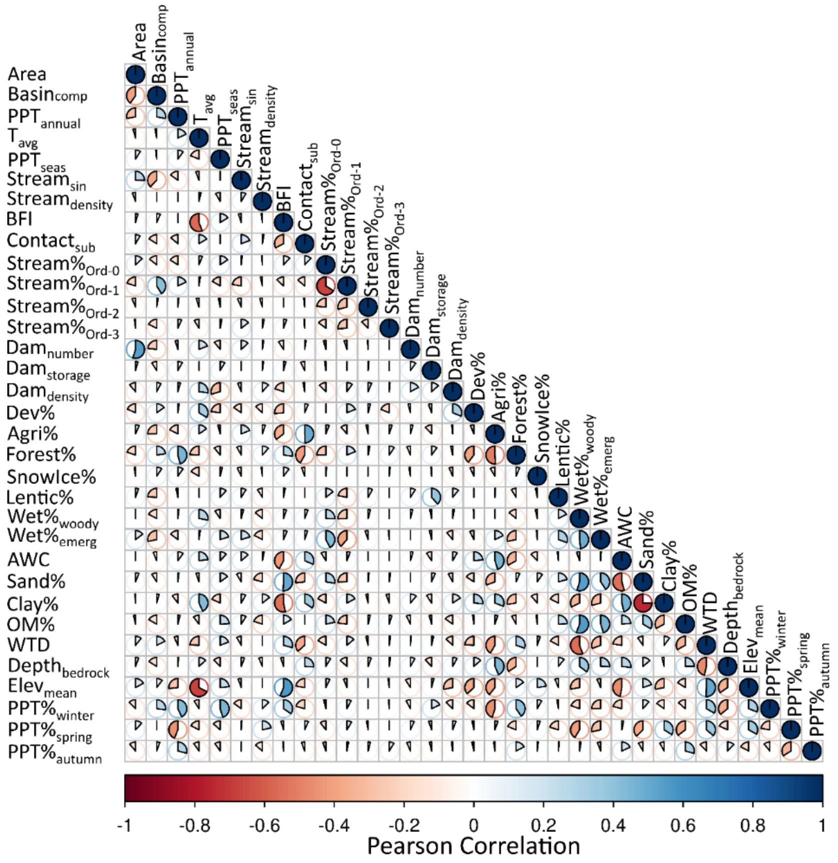

Fig. S10. **Pearson correlation coefficient between the 33 catchment attributes** for 1471 sites. Table S1 contains the full form of abbreviations of the attributes, their units and source.

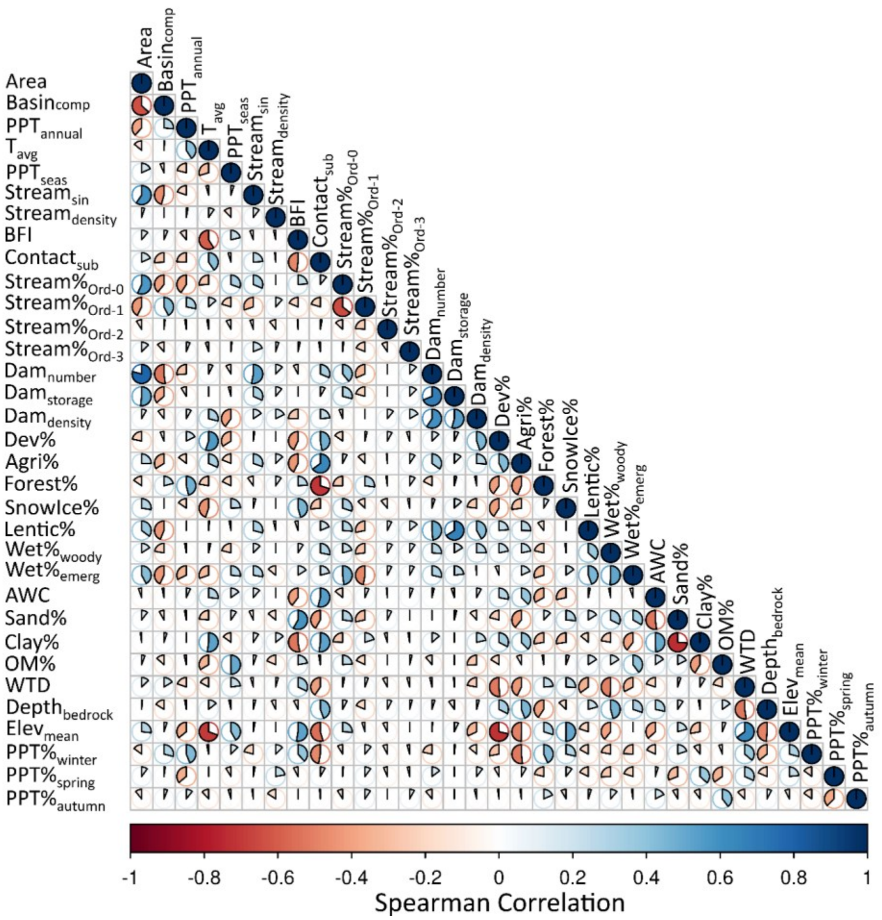

Fig. S11. **Spearman correlation coefficient between the 33 catchment attributes** for 1471 sites. Table S1 contains the full form of abbreviations of the attributes, their units and source.

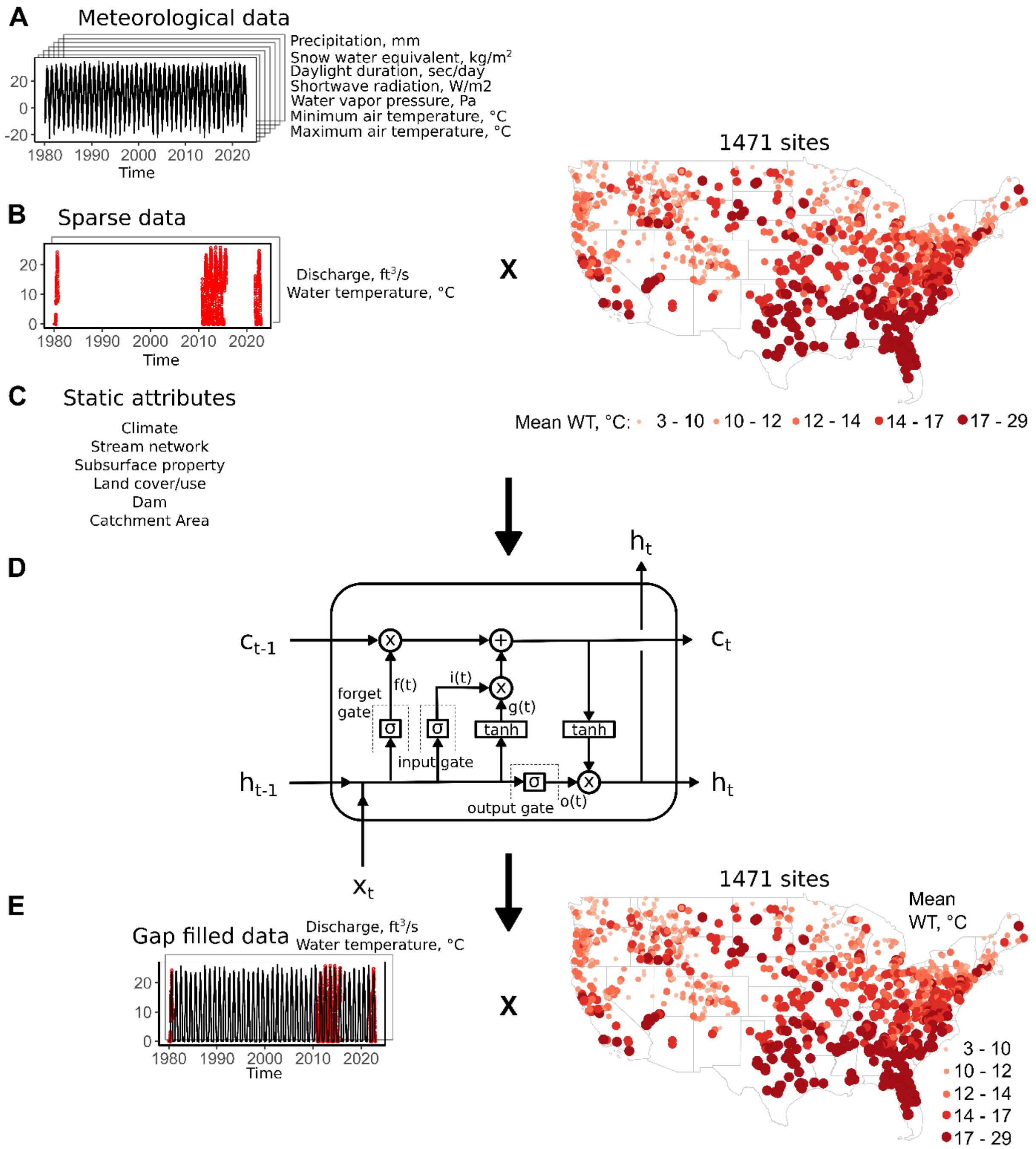

Fig. S12. Gap filling of data using Long Short-Term Memory (LSTM) model. Data from 1471 sites used as input for model include (**A**) daily meteorological data; (**B**) sparse water temperature and discharge observation data; and (**C**) constant catchment attributes; (**D**) LSTM model structure; (**E**) gap filled water temperature and discharge data output by LSTM for 1471 sites.

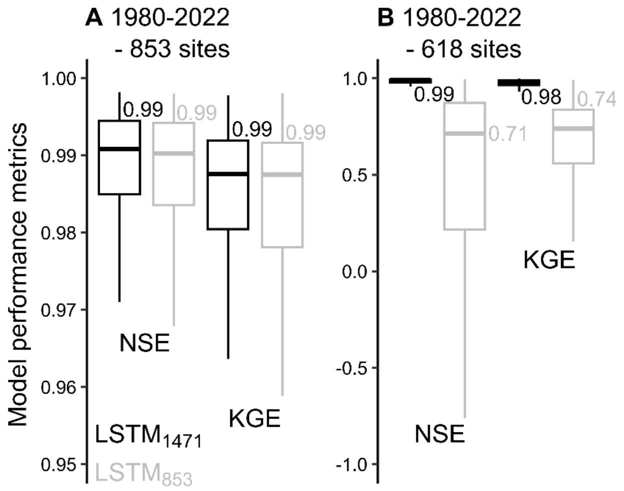

Fig S13. **Comparison of models built with 1,471 sites ($LSTM_{1471}$, black) and with 853 sites ($LSTM_{853}$, grey).** (**A**) NSE and KGE values for daily water temperature predictions of both models for the overlapping 853 sites over 1980-2022; and (**B**) NSE and KGE values for daily water temperature in the 618 sites included in $LSTM_{1471}$ but not in $LSTM_{853}$ over 1980-2022. The comparison indicates that the performance of $LSTM_{853}$ is good in the 853 sites but extrapolation to other sites not included in the training data leads to relatively poor performance. It is therefore better to use all 1,471 sites in training the LSTM model to get better predictions for more sites.

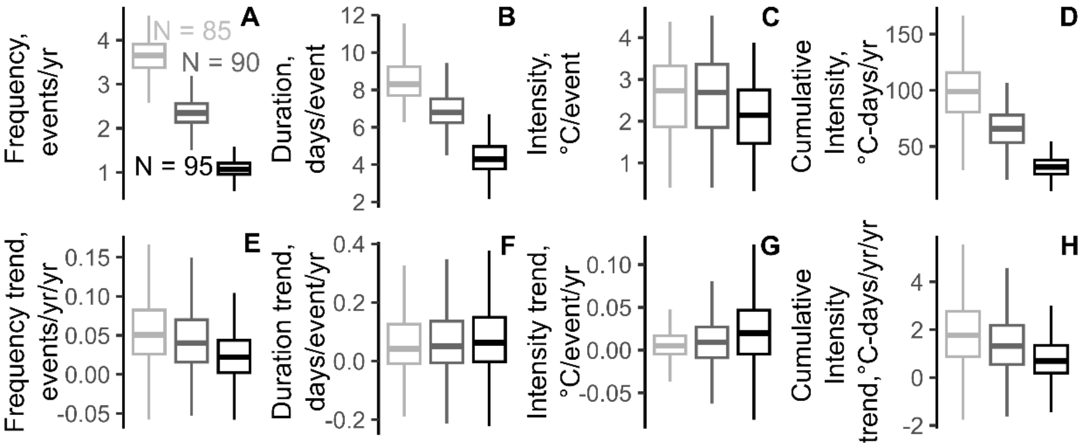

Fig. S14. **Comparison of annual riverine heat wave characteristics and trends under different thresholds of 85th, 90th and 95th percentile.** Distribution of annual (**A**) frequency; (**B**) duration; (**C**) intensity; (**D**) cumulative intensity; trends in annual (**E**) frequency; (**F**) duration; (**G**) intensity; and (**H**) cumulative intensity of 1,276 best performing sites under different thresholds.

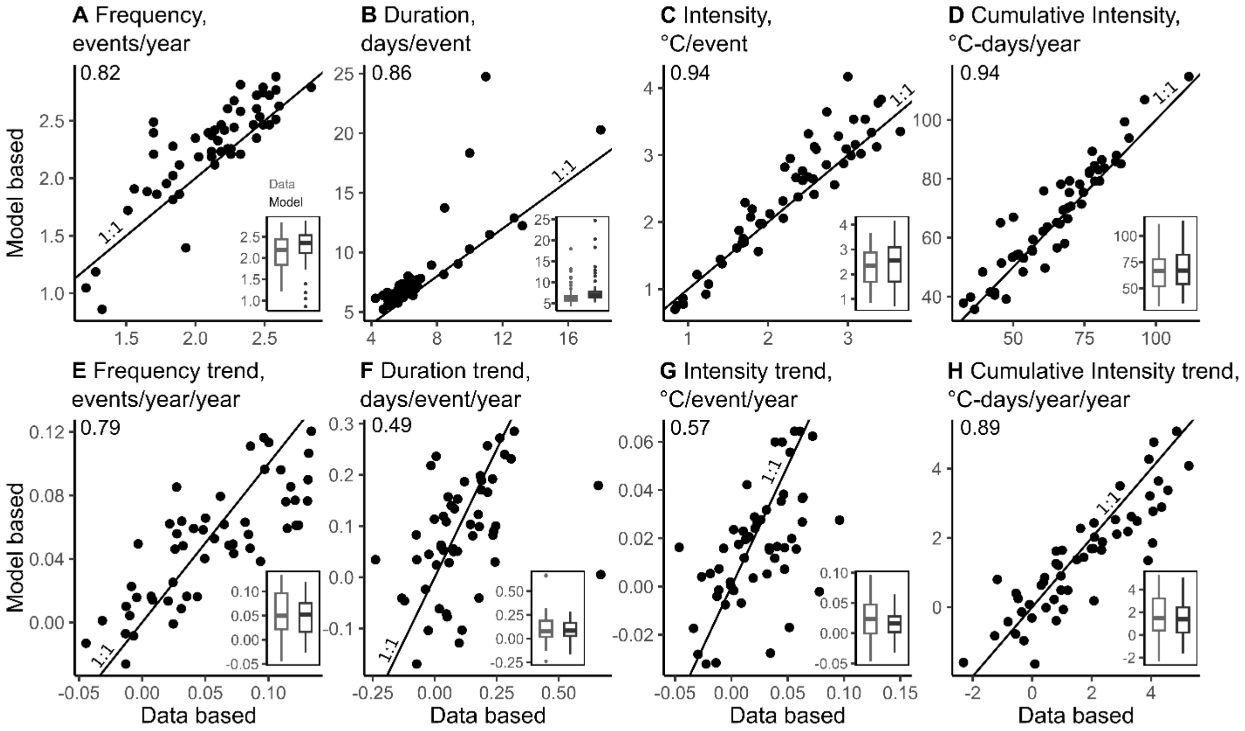

Fig. S15. **Comparison of annual riverine heat wave characteristics and trends based on model versus data.** Comparison of annual (**A**) frequency; (**B**) duration; (**C**) intensity; (**D**) cumulative intensity and trends in annual (**E**) frequency; (**F**) duration; (**G**) intensity; and (**H**) cumulative intensity for 53 sites with most measured data when calculated based on modeled versus measured water temperature data along with their spearman correlation. The numbers inferred from model and data have similar ranges and are highly correlated.

Table S1 The 33 constant catchment attributes (Source: GAGES II) used in the Long Short-Term Memory (LSTM) model, along with their range and median among 1471 sites.

No.	Attributes	Abbreviation	Unit	Range	Median
1	Drainage Area	Area	km^2	1.62 – 49802.27	800.66
2	Basin compactness ratio	$Basin_{comp}$	-	0.34 – 3.82	1.56
3	Mean annual air temperature	T_{avg}	° C	-1.94 – 22.64	9.39
4	Mean annual precipitation	PPT_{annual}	cm/year	20.80 – 415.84	111.55
5	Precipitation seasonality index	$PPT_{seasonal}$	-	0.00 – 0.71	0.12
6	Sinuosity of mainstem stream	$Stream_{sin}$	-	1.01 – 4.86	1.56
7	Stream density	$Stream_{density}$	km/km^2	0.01 – 1.49	0.72
8	Base flow index	BFI	%	6.66 – 87.48	49.96
9	Subsurface flow contact time	$Contact_{sub}$	Days	1.36 – 9538.11	151.17
10	Precent stream of order 0	$Stream\%_{Ord\_0}$	%	0 – 100	1.33
11	Precent stream of order 1	$Stream\%_{Ord\_1}$	%	0 – 100	59.35
12	Precent stream of order 2	$Stream\%_{Ord\_2}$	%	0 – 66.76	17.84
13	Precent stream of order 3	$Stream\%_{Ord\_3}$	%	0 – 46.06	9.58
14	Number of dams	Dam_{number}	Number	0 – 1631	6
15	Dam storage	$Dam_{storage}$	ML/km^2	0 – 3620.76	6.40
16	Dam density	$Dam_{density}$	$no/100km^2$	0 – 28.04	0.56
17	Percent developed area*	Dev%	%	0 – 99.97	5.05
18	Percent agricultural area*	Agri%	%	0 – 93.25	7.18
19	Percent forest area*	Forest%	%	0 – 100	45.00
20	Percent perennial ice/snow cover*	SnowIce%	%	0 – 18.93	0
21	Percent water cover (dam/lake)*	Lentic%	%	0 – 27.80	0.51
22	Percent woody wetland area*	$Wet\%_{woody}$	%	0 – 61.53	0.52
23	Percent emergent wetland area*	$Wet\%_{emerg}$	%	0 – 15.20	0.09
24	Available water capacity	AWC	inches/inches	0.05 – 0.25	0.13
25	Soil sand content	Sand%	%	4.28 – 91.60	33.18
26	Soil clay content	Clay%	%	3.75 – 57.59	21.75
27	Soil organic matter content	OM%	%	0.17 – 21.62	0.84
28	Water table depth	WTD	Feet	0.44 – 6.00	5.35
29	Depth to bedrock	$Depth_{bedrock}$	Inches	17.5 – 65.28	51.03
30	Mean elevation	$Elev_{mean}$	Meters	6.40 – 3600.23	440.01

31	Winter precipitation percent	$PPT\%_{winter}$	%	6.30 – 61.44	22.68
32	Spring precipitation percent	$PPT\%_{spring}$	%	17.71 – 38.55	26.51
33	Autumn precipitation percent	$PPT\%_{autumn}$	%	11.29 – 31.19	23.98

*The landcover characteristics correspond to year 2006

Table S2 Mean (± 1 standard deviation) of annual air and riverine heat wave characteristics and their trends of 1276 sites and among different land uses

	**All**	**Undev**	**Mixed**	**Urban**	**Agri**	**Unit**
**Mean of annual air heat wave:**						
Frequency	4.61 (±0.39)	4.69 (±0.40)	4.57 (±0.38)	4.53 (±0.39	4.34 (±0.32)	events/yr
Duration	4.04 (±0.27)	4.04 (±0.26)	4.04 (±0.27)	4.06 (±0.28)	3.98 (±0.25)	days/event
Intensity	7.66 (±1.17)	7.69 (±1.03)	7.66 (±1.23)	7.38 (±1.20)	8.62 (±1.80)	°C/event
Cumulative intensity	152.40 (±19.71)	157 (±18.50)	149.51 (±19.08)	142.57 (±16.99)	165.80 (±33.01)	°C-days/yr
**Mean of annual riverine heat wave:**						
Frequency	2.34 (±0.33)	2.26 (±0.32)	2.43 (±0.33)	2.28 (±0.22)	2.25 (±0.28)	events/yr
Duration	7.23 (±1.82)	7.21 (±1.91)	7.39 (±1.92)	6.66 (±0.76)	7.60 (±1.21)	days/event
Intensity	2.61 (±0.90)	2.13 (±0.84)	2.90 (±0.81)	3.20 (±0.59)	3.11 (±0.69)	°C/event
Cumulative intensity	65.67 (±16.45)	58.38 (±15.35)	72.46 (±15.74)	67.21 (±11.63)	67.47 (±12.49)	°C-days/yr
**Mean of trend in annual air heat wave:**						
Frequency	0.015 (±0.050)	0.024 (±0.053)	0.009 (±0.046)	0.012 (±0.039)	-0.032 (±0.058)	events/yr/yr
Duration	-0.001 (±0.020)	0.003 (±0.020)	-0.003 (±0.020)	-0.006 (±0.016)	-0.011 (±0.015)	days/event/yr
Intensity	-0.002 (±0.031)	-0.004 (±0.031)	-0.001 (±0.033)	0.000 (±0.019)	-0.007 (±0.028)	°C/event/yr
Cumulative intensity	0.405 (±1.833)	0.603 (±2.073)	0. 304 (±1.582)	0.380 (±1.457)	-1.441 (±2.073)	°C-days/yr/yr
**Mean of trend in annual riverine heat wave:**						
Frequency	0.044 (±0.041)	0.057 (±0.041)	0.034 (±0.039)	0.040 (±0.031)	-0.008 (±0.032)	events/yr/yr
Duration	0.077 (±0.127)	0.116 (±0.132)	0.047 (±0.121)	0.053 (±0.095)	0.004 (±0.063)	days/event/yr
Intensity	0.010 (±0.026)	0.019 (±0.024)	0.002 (±0.025)	0.002 (±0.023)	0.009 (±0.018)	°C/event/yr
Cumulative intensity	1.427 (±1.290)	1.644 (±1.286)	1.263 (±1.293)	1.481 (±1.104)	0.034 (±0.856)	°C-days/yr/yr

Table S3 Relative percentage of sites showing significant positive (>0, $p \leq 0.05$), insignificant changes (~0, $p \leq 0.05$) and significant negative (<0, $p \leq 0.05$) trends in air and riverine heat wave characteristics among 1276 sites and different land uses

	**Air heat waves (%)**				**Riverine heat waves (%)**			
	Frequency	Duration	Intensity	Cumulative Intensity	Frequency	Duration	Intensity	Cumulative Intensity
**All (1276 sites)**								
>0 (p≤0.05)	45	35	35	47	80	62	54	81
~0 (p≥0.05)	27	19	24	21	12	18	18	13
<0 (p≤0.05)	28	46	41	32	8	20	28	6
**Undev (552 sites)**								
>0 (p≤0.05)	52	39	30	49	88	77	72	87
~0 (p≥0.05)	24	24	23	19	9	14	15	10
<0 (p≤0.05)	23	38	47	32	3	8	12	3
**Mixed (552 sites)**								
>0 (p≤0.05)	38	33	39	41	73	49	40	77
~0 (p≥0.05)	31	15	22	26	15	22	19	14
<0 (p≤0.05)	31	52	39	33	12	29	42	9
**Urban (145 sites)**								
>0 (p≤0.05)	49	28	36	61	82	60	41	83
~0 (p≥0.05)	27	17	39	16	12	14	19	14
<0 (p≤0.05)	24	55	26	23	6	26	39	2
**Agri (27 sites)**								
>0 (p≤0.05)	22	11	33	26	22	44	41	26
~0 (p≥0.05)	15	33	19	7	22	15	52	33
<0 (p≤0.05)	63	56	48	67	56	41	7	41

Table S4 Land use classification criteria according to USGS

Land use	Abbreviation	Agricultural cover, %	Urban cover, %
Undeveloped	Undev	≤ 25%	≤ 5%
Urban	Urban	≤ 25%	> 25%
Agricultural	Agri	> 50%	≤ 5%
Mixed	Mixed	Rest	

Table S5 The 19 additional attributes used as candidates for boosted regression tree model to identify important attributes influencing trends in characteristics of riverine heat waves along with their range and median for 1276 sites with best model performance

No.	Attributes	Abbreviation	Unit	Range	Median	Source
1	Mean annual runoff from gap filled daily discharge	Q_{annual}	mm/year	0.72 – 3795.805	398.79	USGS; LSTM
2	Mean stream water/air amplitude ratio	AR	-	0.14 – 1.16	0.84	LSTM (water temperature); Daymet (23) (air temperature)
3	Trend in annual precipitation*	PPT_{trend}	mm/year /year	-12.68 – 70.09	1.76	Daymet (23)
4	Trend in maximum air temperature*	$Tmax_{trend}$	°C/year	-0.05 – 0.09	0.01	Daymet (23)
5	Trend in minimum air temperature*	$Tmin_{trend}$	°C/year	-0.02 – 0.18	0.03	Daymet (23)
6	Trend in snow water equivalent*	SWE_{trend}	kg/m^2/year	-9.72 – 0.94	0.00	Daymet (23)
7	Trend in gap filled daily discharge*	Q_{trend}	mm/year /year	-18.58 – 64.40	0.79	USGS (24); LSTM
8	Trend in annual number of low-flow days**	LFD_{trend}	days/year /year	-6.22 – 6.54	-0.22	USGS(24); LSTM
9	Trend in annual mean daily discharge** during low-flow days	LFQ_{trend}	mm/day/ year	-0.04 – 0.08	0.00	USGS(24); LSTM
10	Trend in annual mean daily water shortage during low-flow days**	LFS_{trend}	mm/day/ year	-0.03 – 0.08	0.00	USGS(24); LSTM
11	Trend in annual stream water/air amplitude ratio**	AR_{trend}	/year	-0.003 – 0.004	0.000	LSTM (water temperature); Daymet(23) (air temperature)
12	Trend in percent water cover**	$Lentic\%_{trend}$	%/year	-0.05 – 0.12	0.00	NLCD (17)
13	Trend in percent developed area**	$Dev\%_{trend}$	%/year	-0.02 – 2.07	0.03	NLCD (17)
14	Trend in percent agricultural area**	$Agri\%_{trend}$	%/year	-1.10 – 0.17	-0.01	NLCD (17)
15	Trend in percent forested area**	$Forest\%_{trend}$	%/year	-3.59 – 1.33	-0.02	NLCD (17)

16	Trend in percent wetland area**	$Wet\%_{trend}$	%/year	-0.14 – 0.02	0.00	NLCD (17)
17	Trend in percent barren area**	$Barren\%_{trend}$	%/year	-0.10 – 0.26	0.00	NLCD (17)
18	Trend in percent shrub cover**	$Shrub\%_{trend}$	%/year	-1.13 – 2.10	0.00	NLCD[3]
19	Trend in percent herbaceous cover**	$Herb\%_{trend}$	%/year	-1.71 – 1.96	0.00	NLCD

**Trends were calculated over daily time series of concerned variable*

***Trends were calculated over annual time series of concerned variable*

Table S6 Summary of mean (± 1 standard deviation or standard error) of annual air, lake, marine and riverine heat wave characteristics and their trends from literature and this work. Please refer to Fig. S5 for visual comparison

**Type**	**Frequency, events/yr**	**Duration, days/event**	**Mean intensity, °C/event**	**Frequency trend, events/yr/yr**	**Duration trend, days/event/yr**	**Mean intensity trend, °C/event/yr**
Air (21)	1.36	4.76	2.5	0.029	0.005	-0.011
Lake (6)	NA	7.7 (± 0.40)	3.7 (± 0.10)	NA	NA	NA
Marine* (5)	2.7 (± 0.28)	14 (± 1.50)	1.3 (± 0.14)	0.09 (± 0.026)	0.23 (± 0.076)	-0.00087 (± 0.0014)
Marine (7)	NA	NA	NA	0.045	0.13	0.0085
Marine (8)	NA	NA	NA	NA	0.141 (± 0.025)	0.0005 (± 0.00006)
Riverine (22)	2	9	1.7	0.06	NA	NA
Air (this work)	4.612 (± 0.392)	4.039 (± 0.267)	7.658 (± 1.170)	0.015 (± 0.050)	-0.001 (± 0.020)	-0.002 (± 0.031)
Riverine (this work)	2.336 (± 0.327)	7.235 (± 1.821)	2.606 (± 0.903)	0.044 (± 0.041)	0.077 (± 0.127)	0.010 (± 0.026)

***Category I alone**

Table S7 Spearman and Pearson correlation of annual riverine heat wave characteristics and trends when 85th and 95th thresholds are used compared to 90th percentile

Riverine heat wave	85th vs 90th percentile		95th vs 90th percentile	
	Spearman correlation	Pearson Correlation	Spearman correlation	Pearson Correlation
Annual frequency	0.83	0.86	0.72	0.73
Annual duration	0.88	0.95	0.8	0.86
Annual intensity	0.98	0.99	0.95	0.95
Annual cumulative intensity	0.98	0.98	0.94	0.94
Trend in annual frequency	0.88	0.91	0.83	0.84
Trend in annual duration	0.88	0.89	0.85	0.84
Trend in annual intensity	0.79	0.8	0.75	0.73
Trend in annual cumulative intensity	0.96	0.97	0.9	0.92

Table S8 Spearman and Pearson correlation between riverine heat wave characteristics and their trends among 53 sites with most data when calculated based on measured and modeled data

Riverine heat wave	Spearman correlation	Pearson Correlation
Annual frequency	0.82	0.84
Annual duration	0.86	0.82
Annual intensity	0.94	0.94
Annual cumulative intensity	0.94	0.94
Trend in annual frequency	0.79	0.80
Trend in annual duration	0.49	0.40
Trend in annual intensity	0.57	0.56
Trend in annual cumulative intensity	0.89	0.88

SI References

1. M. G. Kendall, Rank correlation methods. (1948).
2. H. B. Mann, Nonparametric tests against trend. *Econometrica: Journal of the econometric society*, 245-259 (1945).
3. S. K. Patakamuri, N. O'Brien, M. S. K. Patakamuri, Package 'modifiedmk'. *Cran. R-project* (2020).
4. A. J. Hobday *et al.*, Categorizing and naming marine heat waves. *Oceanography* **31**, 162-173 (2018).
5. S. M. Chiswell, Global Trends in Marine Heat waves and Cold Spells: The Impacts of Fixed Versus Changing Baselines. *Journal of Geophysical Research: Oceans* **127**, e2022JC018757 (2022).
6. R. I. Woolway *et al.*, Lake heat waves under climate change. *Nature* **589**, 402-407 (2021).
7. E. C. J. Oliver *et al.*, Longer and more frequent marine heat waves over the past century. *Nat Commun* **9**, 1324 (2018).
8. Y. Yao, C. Wang, Y. Fu, Global Marine Heat waves and Cold-Spells in Present Climate to Future Projections. *Earth's Future* **10**, e2022EF002787 (2022).
9. R. A. Peterson, M. R. A. Peterson, Package 'bestNormalize'. *Normalizing transformation functions. R package version* **1** (2020).
10. K. Sadayappan, D. Kerins, C. P. Shen, L. Li, Nitrate concentrations predominantly driven by human, climate, and soil properties in US rivers. *Water Res* **226** (2022).
11. J. H. Friedman, Greedy function approximation: A gradient boosting machine. *Ann Stat* **29**, 1189-1232 (2001).
12. C. Kelleher *et al.*, Investigating controls on the thermal sensitivity of Pennsylvania streams. *Hydrol Process* **26**, 771-785 (2012).
13. J. Wade, C. Kelleher, D. M. Hannah, Machine learning unravels controls on river water temperature regime dynamics. *J Hydrol* **623**, 129821 (2023).
14. S. Hochreiter, J. Schmidhuber, Long short-term memory. *Neural computation* **9**, 1735-1780 (1997).
15. A. J. Hobday *et al.*, A hierarchical approach to defining marine heat waves. *Progress in oceanography* **141**, 227-238 (2016).
16. D. K. Hare, A. M. Helton, Z. C. Johnson, J. W. Lane, M. A. Briggs, Continental-scale analysis of shallow and deep groundwater contributions to streams. *Nat Commun* **12** (2021).
17. U. S. G. Survey (2025) Annual NLCD (National Land Cover Database)—The next generation of land cover mapping: U.S. Geological Survey Fact Sheet 2025–3001.
18. D. Berrar, "Cross-Validation" in Encyclopedia of Bioinformatics and Computational Biology. (Academic Press, Oxford, 2019), https://doi.org/10.1016/B978-0-12-809633-8.20349-X, pp. 542-545.
19. D. Berrar (2019) Cross-validation.
20. T. Chen, T. He, M. Benesty, V. Khotilovich, Package 'xgboost'. *R version* **90** (2019).
21. Y. Zhang, Q. Li, Y. Ge, X. Du, H. Wang, Growing prevalence of heat over cold extremes with overall milder extremes and multiple successive events. *Commun Earth Environ* **3**, 1-13 (2022).
22. S. J. Tassone *et al.*, Increasing heat wave frequency in streams and rivers of the United States. *Limnol Oceanogr Lett* **8**, 295-304 (2023).
23. M. M. Thornton, R. Shrestha, Y. Wei, P. E. Thornton, S. C. Kao, Daymet: Daily Surface Weather Data on a 1-km Grid for North America, Version 4 R1. http://dx.doi.org/10.3334/ORNLDAAC/2129.
24. USGS (2024) National water information system data available on the world wide web (USGS water data for the nation). (USGS Surface-Water Annual Statistics for Wisconsin).
